# A novel in-situ-process technique constructs whole circular cpDNA library

**DOI:** 10.1186/s13007-023-01126-7

**Published:** 2024-01-03

**Authors:** Qiang Zhou, Xianlong Ding, Hongjie Wang, Zunaira Farooq, Liang Wang, Shouping Yang

**Affiliations:** Key Laboratory of Biology and Genetics Improvement of Soybean, Ministry of Agriculture of the People’s Republic of China, Zhongshan Biological Breeding Laboratory (ZSBBL), National Innovation Platform for Soybean Breeding and Industry-Education Integration, State Key Laboratory of Crop Genetics & Germplasm Enhancement and Utilization, National Center for Soybean Improvement, Jiangsu Collaborative Innovation Center for Modern Crop Production, Soybean Research Institute, College of Agriculture, Nanjing Agricultural University, Nanjing, China

**Keywords:** In-situ-process, In-situ lysis, In-situ substitute/ligation, BAC library, Circular cpDNA, Chloroplast

## Abstract

**Background:**

The chloroplast genome (cp genome) is directly related to the study and analysis of molecular phylogeny and evolution of plants in the phylogenomics era. The cp genome, whereas, is highly plastic and exists as a heterogeneous mixture of sizes and physical conformations. It is advantageous to purify/enrich the circular chloroplast DNA (cpDNA) to reduce sequence complexity in cp genome research. Large-insert, ordered DNA libraries are more practical for genomics research than conventional, unordered ones. From this, a technique of constructing the ordered BAC library with the goal-insert cpDNA fragment is developed in this paper.

**Results:**

This novel in-situ-process technique will efficiently extract circular cpDNA from crops and construct a high-quality cpDNA library. The protocol combines the in-situ chloroplast lysis for the high-purity circular cpDNA with the in-situ substitute/ligation for the high-quality cpDNA library. Individually, a series of original buffers/solutions and optimized procedures for chloroplast lysis in-situ is different than bacterial lysis in-situ; the in-situ substitute/ligation that reacts on the MCE membrane is suitable for constructing the goal-insert, ordered cpDNA library while preventing the large-insert cpDNA fragment breakage. The goal-insert, ordered cpDNA library is arrayed on the microtiter plate by three colonies with the definite cpDNA fragment that are homologous-corresponds to the whole circular cpDNA of the chloroplast.

**Conclusion:**

The novel in-situ-process technique amply furtherance of research in genome-wide functional analysis and characterization of chloroplasts, such as genome sequencing, bioinformatics analysis, cloning, physical mapping, molecular phylogeny and evolution.

**Supplementary Information:**

The online version contains supplementary material available at 10.1186/s13007-023-01126-7.

## Background

The cp genome is highly plastic and is a heterogeneous mixture of sizes and physical conformations. The ploidy level within the nucleus is relatively stable during leaf development, whereas the chloroplast nucleoids are unstable [1, 2]. The cp genomes typically assemble as genome-sized circular DNA.

However, it is now widely known that cpDNAs have more complex and dynamic conformations than cpDNA assemblies suggest: monomer, dimer, trimer, tetramer forms, linear-branched forms and atypical cpDNA molecules [3–7]. As described by Lampapa and Kuroiwa, the cpDNA content can alter throughout leaf development, and the amount and structure are impacted by light and genotype [8, 9]. Shaver and Oldenburg have assumed a syllogistically coherent period, which is classified as the plastids (I), nucleoids (II), and cpDNA (III), and 2 years afterward, they proposed the content of subgenomic/monomer cpDNA is associated with the stage of chloroplast development [10–12].

In-situ lysis for PFGE. David C. S. and Charles F. C. devised a new type of gel electrophoresis technology known as Pulsed-Field Gel Electrophoresis (PFGE) and Gel Insert to separate the higher molecular weight DNA molecules with a more excellent resolution for large DNA without breakage [13]. It can separate the DNA molecules up to 2000 Kb with resolutions exceeding the logarithmic molecular weight dependence of conventional electrophoresis. Following this study, Lex H. T. V at the Department of Molecular Biology Netherlands Cancer Institute fractionated the chromosome-sized DNA molecules of *Trypanosoma brucei*, strain 427, using Pulsed field gradient gel electrophoresis. It was consequently of extreme urgency and significance for the researchers who concentrated on chromosome-sized DNA molecules with the units Kb and Mb.

In 1990 and 1991, Bendich A. J. and Smith S. B. deployed the In-situ lysis for PFGE approaches to cp genome conformations study, backed up the widely held belief at the time that cp genome occurs in chloroplast as circular molecules of genome size [4, 14]. But as far as the writer knows, Xing-wang D’s work first used the In-situ lysis for PFGE approaches in cp genome structural research [3]. In 1995, an oppositely purposeful protocol was published that described the lysis and PFGE approaches for eliminating the cp genome from plant cells to extract the nuclear DNA [15]. In parallel, the In-situ lysis for PFGE approaches has been systematically described and improved [5]. After the in-situ chloroplast lysis used for cp genome structural research, it has not been used for the circular cpDNA extraction field till now. In the meantime, the reagents and the steps which are unaltered quoted from the in-situ bacterial lysis method, are inefficient in chloroplast lysis, because of the difference between the peptidoglycan/mucopeptide of the bacterial cell wall and lipid bilayer of chloroplast membrane.

The historical course of cpDNA extraction method. Among the developed cpDNA extraction techniques with an aqueous phase (like the CTBA method and alkaline lysis) caused the fragmentation of the circular cpDNA and a few residual non-target DNA impurities are inevitable, the high-quality circular cpDNA is not perfect in the conventional extraction method. The method in the choiceness article of cp genome extraction between 1963 and 2021 is chronicled in Additional file 2: Table S1[3–6, 10, 14, 16–49]. Updating the techniques in the cp extraction method is the consequence of the consistent efforts of the industrious scientific research people, although the space is constrained to be outlined.

BAC library. the large-insert cloning capacities (over 300 kb) of BAC have reduced the colonies number that need to represent a complete DNA library of an organism [50, 51]. BAC is stable in the bacterial host and low in chimeric clones, and their DNA is readily purified from the host DNA. All of which is crucial to genomics research. Large-insert, ordered DNA libraries are more practical for genomics research than conventional, unordered ones. The clones of interest could be quickly obtained from the well-organized library without needing the time-consuming procedure for the purification of positive clones [52]. For genomics research, large-insert, ordered BAC has made it achievable to create complicated whole-genome physical maps [53] and to map genes and QTL utilizing map-based cloning [54, 55]. The construction of large-insert, ordered DNA libraries is a necessary resource for large-scale genome sequencing, and they enable genome-wide functional analysis and large-scale gene discovery, cloning, and characterization [56].

In-situ substitute/ligation protocol in this experiment has been developed for a high ligation efficiency in vector/insert recombinant DNA while ensuring the integrity of the cpDNA sequence within the length of 90.0 kb. Based on this protocol, we have successfully constructed the goal-insert, ordered cpDNA library to replace the conventional, unordered ones that the cpDNA fragment is random from the conventional extraction method. The in-situ substitute/ligation protocol can not be limited to cpDNAs, it is also used for the other nucleic acid sequences to produce the kilobase-sized, goal-clonable DNA fragments and construct the goal-insert, ordered DNA libraries.

## Materials and methods

### Materials

The materials are listed in Table 1.

**Table 1:** Experimental material

Cultivar	Common name	Latin name	Family	Cotyledon
Bai nong 207	Wheat	*Triticum aestivum*	Poaceae (grass)	Monocot
Su ke yu 208	Maize	*Zea mays*	Poaceae (grass)	Monocot
Williams 82	Soybean	*Glycine max*	Fabaceae (pea)	Eudicot
Su pi 3	Barley	*Hordeum vulgare*	Poaceae (grass)	Monocot

### Reagents, equipment

5 mM/10 mM Tris-HCl (pH = 7.5), autoclave, stored at 4 °C. Low-melting-point agarose (LMP, 2%/1%). Glycogen (Inert co-precipitant, ThermoFisher R 0561). 0.2% BSA, stored at 4 °C. Exonuclease V (RecBCDase, BioLabs M 0345 S). Lambda PFG Ladder (BioLabs N 0341 S). Phanta HS Super-Fidelity DNA Polymerase (Vazyme, P 502—d 1). ElectroMAX™ DH10B Cells (ThermoFisher 18290015). ET-M-D buffer: 0.6 M d-mannitol, 0.05 M MES, 0.05 M Tris, 75% ethanol, pH to 6.5 with HCl/NaOH, must be made fresh before use and stored at room temperature. W5-3 solution: 0.1 M NaCl, 0.15 M CaCl_2_, 0.005 M KCl, 0.0015 M MES, 0.1 M Tris, 0.05 M EDTA.Na_2_, pH to 7.5 with NaOH, autoclave, stored at 4 °C. 0.5%/2% low-melting-point agarose: the solvent is 10 mM Tris-HCl, 0.5% and 2% (W/V) LMP agarose, stored at 65 °C. 1% low-melting-point agarose: the solvent is 0.5 × TBE (from 5 × TBE), 1% (W/V) LMP agarose. 1 × TE buffer: 0.05 M EDTA, 0.075 M Tris, pH to 7.5 with NaOH, autoclave, stored at 4 °C. Et-S buffer: 0.2 M NaCl, 0.05 M MES, 0.5% SDS, 0.25 M Tris, 0.4% Tween 20, 40% ethanol, pH to 7.5 with HCl/NaOH, must be made fresh before use. pK solution: 20 mM Tris-HCl, 1 mM CaCl_2_, pH to 8.0 with HCl/NaOH, autoclave, stored at 4 °C. NDS-1 buffer: 0.1 M Tris, 0.05 M EDTA, 1% SDS, pH to 8.0 with HCl/NaOH. Filtration by 0.22 μm, stored at − 20 °C, before use, adds Proteinase K to 2 mg/mL and Spermidine trihydrochloride to 20 mM. X solution: 3% glucose, 0.2% PVP, 0.2 mM Tris-HCI, pH to 8.0 with HCl/NaOH, autoclave (105 °C/5 min), stored at 4 °C.

Illumination incubator (Panasonic MLR-352-PC/MLR-352 H-PC). Electric-heated thermostatic water bath (JULABO PURA 22). Oscillating constant temperature metal bath (BIOER, BILON BL-100 D). MCE membrane (MF-Millpore, VSWP 04700). Centrifuge (Sigma 10033, ThermoFisher 75004380). Electrophoresis apparatus (Bio-rad CHEF-DRII, Bio-rad 1658033). Supporting tray (Merckmillipore 71551, Merckmillipore 71504). PCR Thermocycle Instrument (Bio-rad 1861096). Gel Imaging & Analysis System (Tanon-2500 B). Electron microscope (Olympus-cx 31). Ultraviolet spectrophotometer (ThermoFisher-NanoDrop One^C^). Gene Pulser Xcell (Bio-rad 1652660). Nucleic Acid Analyzer (ThermoFisher, NanoDrop One).

### Protocol for the in-situ-process

**Fig. 1:**
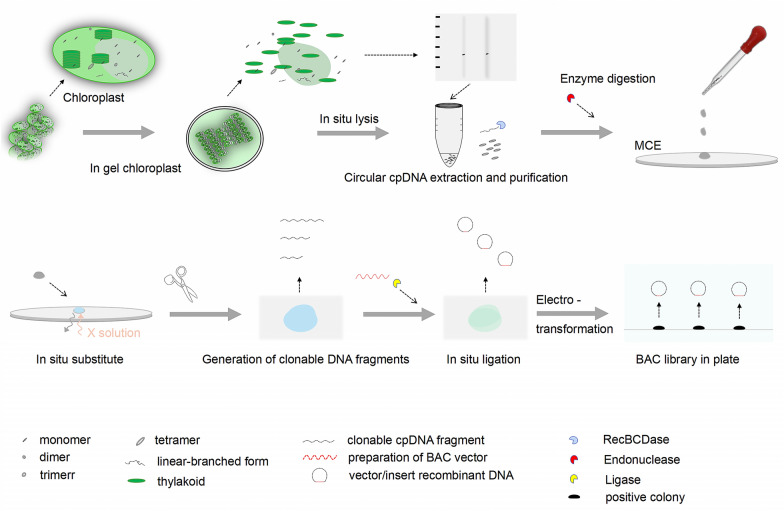
Workflow of the in-situ-process technique

In this study, we extract the circular cpDNA to construct the cpDNA libraries by the in-situ-process (Fig. 1). The exact details of this procedure are as follows: the crucial aspects connected to the experimental outcomes are highlighted with the phrase attention.

### In-situ chloroplast lysis

Crude chloroplast preparation. The seeds are germinated in a greenhouse using vermiculite for 3–5 days, and the seedlings are then carefully moved into the nutrient solution (Hoagland) to promote the growth of sensitive, low-carbohydrate leaves.

Attention ① The hydroponic data: Wheat (14 h light at 28 °C, 10 h dark at 25 °C, humidity 70%, replace the nutrient solution every 7 days for 30–35 d. Additional file 1: Fig. S1a2); Maize (12 h light at 30 °C, 12 h dark at 23 °C, humidity 70%, replace the nutrient solution every 7 days for 30–35 d. Additional file 1: Fig. S1a3); Soybean (14 h light at 25 °C, 10 h dark at 21 °C, humidity 70%, replace the nutrient solution every 7 days for 30–35 d. Additional file 1: Fig. S1a4); Barley (14 h light at 28 °C, 10 h dark at 25 °C, humidity 70%, replace the nutrient solution every 7 days for 30–35 d. Additional file 1: Fig. S1a1). ② Hydroponics reduces starch accumulation in chloroplast in that not easy form the intramembrane high pressure, help for chloroplast extraction. ③ Cell disintegration and the succeeding isolation of chloroplast should be carried out within a minimal time limit.

Immersing 15 g tender leaves into 100 mL of ET-M-D buffer 10–20 min. Clean the wet-packed leaves by the W5-3 solution, grind the leaves with a tissue homogenizer, slowly-mix until fluffy after pouring the homogenized tissue (grinding conditions: Blender, 5 × 90 s at 1800 rpm) into the Erlenmeyer flask with 300 mL W5-3 solution.

Attention ① sampling stage: Wheat, 3–4 leaf stage; Maize, V2–V3 stage; Soybean, V3–V4 stage; Barley, 3–4 leaf stage. ② A light green color appears in the buffer after the leaves are macerated by ET-M-D buffer. The sample types determined the immersing time: Wheat 20 min; Maize 10 min; Soybean 10 min; Barley 20 min. Notably, be vigilant about intimal rupture caused by timing out. Always use freshly made ET-M-D buffer for the sample maceration. ③ Keep the samples on ice during all operations, including transit to and from the centrifuge. Do not lyophilize the samples by flash-freezing, such as inserting the sample into liquid nitrogen and cryogenic storage (− 20 °C) or dry ice. ④ All equipment is immaculate. Unless otherwise specified, all reagent is pre-cooled and held in cold water. ⑤ Grinding conditions have a marked impact on chloroplast isolation. ⑥ Homogenizing the tissues in liquid nitrogen is firmly not recommended because destroying the intact chloroplast membrane is easier.

Numerous non-target contaminants, including intact cells, living leaf tissue, and other detritus, are present in the disintegrating cell solution. These are initially eliminated by filtering via a fine-mesh nylon gauze filter (100 mesh). Suspended chloroplasts are withdrawn using a serological pipette after the low-speed centrifugation (30 min at 100×*g* and 4 °C). Centrifugation for 10 min at 500×*g* at 4 °C, aspirate the supernatant, and centrifuge the supernatant for 15 min (5 min × 3) at 1200×*g* and 4 °C. Carefully resuspend the pellet with 3–5 mL 5 mM Tris-HCl for a highly turbid solution (≧ 2.5 × 10^7^/mL; Additional file 1: Fig. S1b).

Attention ① The centrifugal condition relies on the size of chloroplasts: Wheat, Maize and Barley are 15 min (5 min × 3) at 1500×*g*/4 °C; Soybean is 15 min (5 min × 3) at 1200×*g*/4 °C. Researchers revealed that numerous short-time centrifugations ((1200×*g*/5 min) × 3 times) are more effective than one long treatment employing continuous centrifugation (1200×*g*/15 min). ② Keep the pellet from being disturbed before resuspending the pellet ③ Soft rubbing the centrifuge tube on ice is advised; do not tremor the centrifuge tube.

#### In-gel chloroplast lysis

**Fig. 2:**
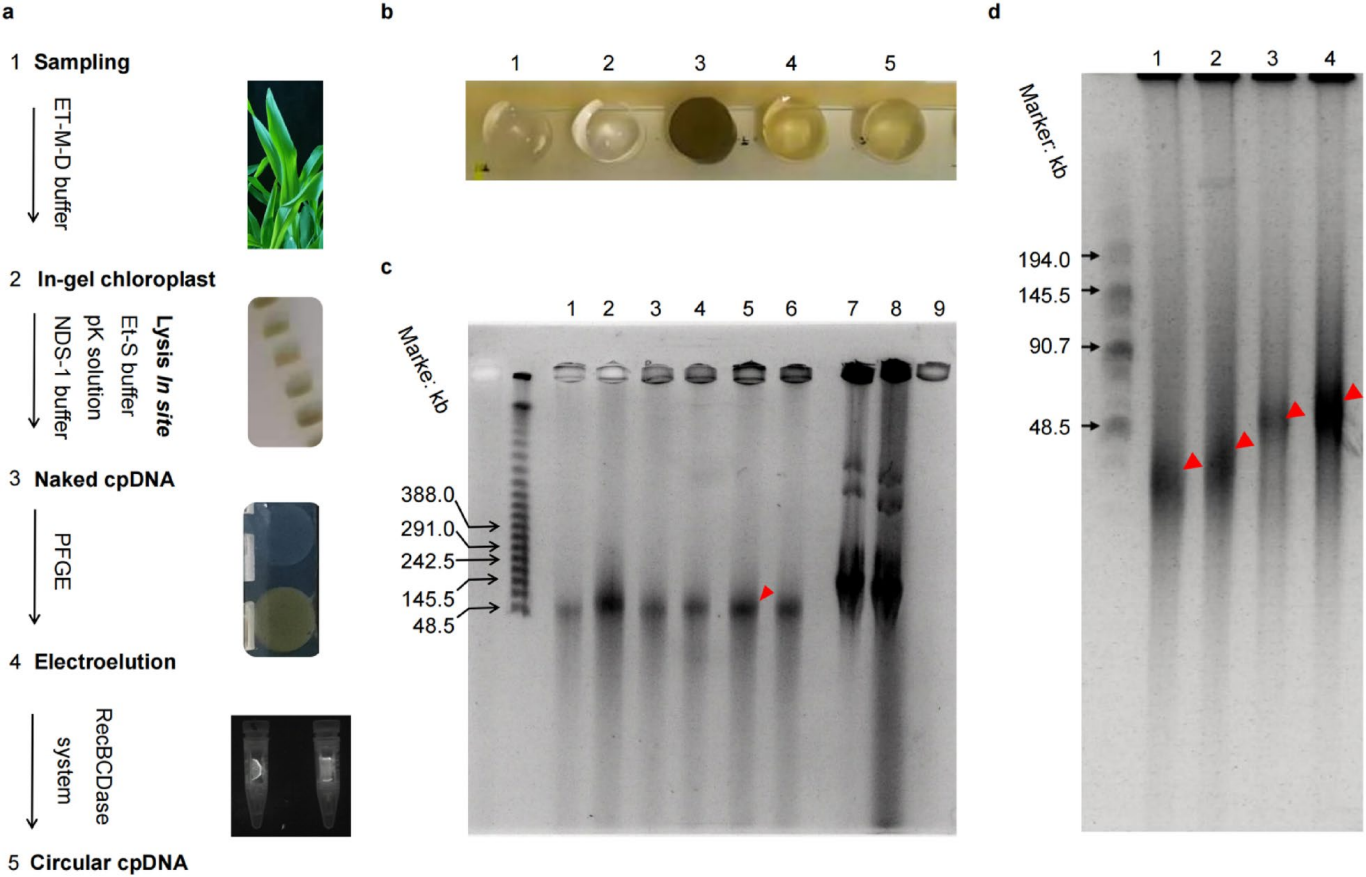
In-situ chloroplast lysis. **a** Illustration of in-situ chloroplast lysis. **b** In-gel chloroplast and its lysis. The Et-S buffer at 35 °C/2.5 h lysed labels 1 and 2 representing the gel blank control −ck (no chloroplast). Label 3 designates the un-lysed in-gel chloroplast control (ck). Labels 4 and 5 depict in-gel chloroplast lysis by Et-S buffer at 35 °C/2.5 h. **c** Lysate efficacy of the Et-S buffer in the In-gel chloroplast. Comparison of the Et-S buffer’s ability to process in-gel chloroplast by a temporal gradient at 35 °C before the pK buffer washed the agarose matrix and the NDS-1 buffer released the histone of cpDNA. The lysate efficacy of the Et-S buffer with the temporal gradient (0.5 h, 1 h, 1.5 h, 2 h, 2.5 h, 3 h) from lane 1 to lane 6 that shown in an autoradiogram from 1% PFGE-gel analysis (Reverse Voltage Gradient: 4.5 V/cm, Int. Sw. Tm: 60 s, Fin. Sw. Tm: 90 s, included angle: 120°, Run Time: 24 h, Ramping factor: a=: ENTER). The lanes (7, 8), as the +ck, were the Et-S buffer lysed the In-gel DH 5α (*E. coli*) for 2.5 h at 35 °C. Lane 9, as the −ck, was the chloroplast gel without lysis buffer for 2.5 h at 35 °C. The results demonstrate that the Et-S buffer has the best lysate effectiveness in In-gel chloroplast (Lane 5, red arrowhead). The HMW lambda ladder is loaded into the far-left corner. **d** Analysis of crop's in-situ chloroplast lysis by PFGE-gel (Reverse Voltage Gradient: 6 V/cm, Int. Sw. Tm: 1 s, Fin. Sw. Tm: 25 s, included angle: 120°, Run Time: 16 h, Ramping factor: a=: ENTER). The circular cpDNA cannot be separated as discrete bands under PGFE conditions, showing the target band (red arrowhead) in PFGE-gel. Lane 1, Wheat circular cpDNA. Lane 2, Maize circular cpDNA. Lane 3, Soybean circular cpDNA. Lane 4, Barley circular cpDNA. The HMW lambda ladder is loaded into the far-left corner. Attention Circular DNA size cannot be marked up with a linearized DNA ladder because of the circular topology properties, whereas it can serve as a relative reference standard

Equilibrating the turbid chloroplast suspension to 45 °C/25 s and rapidly gently resuspended into the equivalent amount of 2% LMP agarose, and then aliquoted into a 200 μL mold using a wide-bore pipette tip. Transfer the in-gel to 50 mL 1 × TE buffer when the bubble-free agarose completely solidifies (Fig. 2b).

Attention ① The test shows a satisfactory embedding effect when the chloroplast suspension is pre-warmed for 25–30 s. Do not exceed the specified time limit, to preserve the intact chloroplasts in the agarose matrix. ② The experimenter should slowly and quickly remix the chloroplast suspension and 2% LMP agarose. To protect the chloroplast membrane, shake the blending meticulously and gradually. We advised experimenter to gently waggle in either a clockwise or anticlockwise direction three to five times only. Avoid prolonged mixing, quick mixing, and shock. They may all cause the chloroplast membrane to be ruptured during embedding. ③ 2% (wt/vol) LMP agarose is melted in 10 mM Tris-HCl (pH = 8.0) and stored at 65 °C.

Washing the in-gel with 30 mL 1 × TE buffers at 35 °C/0.5 h in a thermostatic oscillator by a minimal stirring speed, and then incubate the in-gel in 30 mL Et-S lysis buffer at 35 °C/2.5 h (Fig. 2b4, 5). Replacing the Et-S buffer with the equal volume pK solution to incubate at 35 °C/10 min. Rinsing the in-gel thrice in 30 mL of ice-cold pK solution and shake it now and then until the in-gel settled to the bottom. The in-gel is placed into 30 mL NDS-1 buffer for 10–12 h at 4 °C, change the NDS-1 buffer 3 times during this period. Proceeding the NDS-1 buffer to room temperature (~ 5 min) and incubating for 9 min at 50 °C. After the in-gel chloroplast has been lysed, put the in-gel on ice-cold 0.5 × TBE buffer and shake now and then. Packing the in-gel into the suitable hole that was previously cut in the 1% pulsed field-certified agarose (Fig. 2c). 0.5% LMP agarose was used as bubble-free glue-sealing to fill the crevices between the agarose matrix and the hole.

Attention ① Light green or a yellowish appeared with occasional mixing at the in-gel after the chloroplast lysed. Throughout the in-gel chloroplast lysis procedures, keep the agarose matrix intact and all the buffers with pH < 8.0. ② The in-gel in pK solution can be stored at 4 °C for at least one week without substantial DNA degradation. When loading the in-gel into the NDS-1 buffer, keep it at 4 °C. The proteinase K would move freely into the agarose matrix and not inactivate. ③ Setting the condition with 50 °C/9 min for the NDS-1 buffer-agarose system is favored to liberate the circular cpDNA in the agarose matrix. Time out will damage the DNA structure.

#### Circular cpDNA extraction and purification

After the chloroplast lyse within the in-gel, the circular cpDNA is released using 1% PFGE-gel, and the details about PFGE conditions are provided in the legends (Fig. 2c, d). Utilize the Dialysis tube to recover the circular cpDNA from unstained gel slices that cutting from the 1% PFGE-gel. The electroelution conditions: 90 V electric current with 5 mM Tris-HCl running buffer for 45 min until the circular cpDNA exits the gel slice. Remove the gel slice within the Dialyzer tube and reverse the polarity of the electric current for 90 s to release the circular cpDNA upside the membrane. Collecting the electroeluent into 1.5 mL RNase-free Microfuge Tubes by using a wide-bore pipette tip. Centrifuging for 1000×*g*/1 min to remove the residue of gel slices (Additional file 1: Fig. S2b).

Attention ① Using 5 mM Tris-HCl as the running buffer in electroelution. ② Filling 5 mM Tris-HCl running buffer into the Dialysis tube that held the unstained gel slices. Avoiding the bubbles in the tube, as they interfere with electroelution. The optimal electroelution time must be tested for each sample and gel concentration.

Trapping circular cpDNA from the electroeluent. Proportioning the reagents to the electroeluent according to the table order (Table 2). Placing the mix at − 20 °C (≧ 3 h or overnight). Centrifugation for 30 min at 16,000×*g* at 4 °C, carefully remove the supernatant without disrupting the pellet. Add 200 μL of 75% ethanol and centrifuge at 4 °C for 20 min at 16,000×*g*. Remove as much of the remaining ethanol as possible and air-dry the pellet at room temperature and darkness for about 10 min. Dissolve the pellet with DNase/RNase-Free ddH_2_O and adjust the nucleic acid concentration for ~ 20 ng/μL.

**Table 2:** Glycogen for trapping the circular cpDNA

Reagent	Volume (μL)
Glycogen (20 μg/μL)	Final 0.1 μg/μL concentration
Sodium acetate (3 M)	0.1 × (volume of electroeluate)
Ethanol (100%)	2.5 × (volume of electroeluate + sodium acetate)

The overall linear nucleic acid impurities in the trapped solution of circular cpDNA are digested by the RecBCDase system (Table 3). Dividing the 50 μL RecBCDase solution to 10 μL (5 × 10 μL) and incubate in a thermocycler for the multi-step process: 37 °C/10 h, 70 °C/10 min, 4 °C/∞, heated lid set to 85 °C, − 20 °C storage.

**Table 3:** RecBCDase system

Reagent	Volume (μL) per reaction	Final concentration
NEB (4)	5	1×
ATPs (10 mM)	15	3 mM
0.2% BSA	2.5	0.01%
RecBCDase	2	2 U
cpDNA (~ 20 ng/μL)	Variable	10 ng/μL
ddH_2_O	Variable	For 50 μL

Attention ① Slowly remix the mixture thoroughly after adding glycogen, sodium acetate, and ethanol to the electroeluent, the solution may become viscous. Avoid foaming that caused the nucleic acid chain rupture. ② The circular cpDNA solution can be stored at − 20 °C for at least 6 months without substantial DNA degradation.

### In-situ substitute/ligation

Preparation of the vector. 500 ng modified plasmid within the 50 μL reaction is digested by 5 U PmeI and 15 U alkaline phosphatase at 37 °C/20 min and purified by gel extraction after electrophoresis.

Attention Both complete digestion and full dephosphorylation are required for the pBeloBAC11-MCS(+). Nonetheless, caution must be taken to prevent excessive digestion and dephosphorylation of the vector DNA. The freshly created vector produced a higher effective ligation than the stored vectors.

#### Generation of clonable DNA fragments

Mono-restriction endonuclease digestion. The circular cpDNA of Wheat, Maize, Soybean, and Barley is digested using the mono-restriction endonucleases SrfI, BssHII, NaeI, and SacII, respectively. The enzyme-digested fragments spanned from 27.2 to 90.0 kb (Additional file 1: Figs. S3–S6). 100 ng circular cpDNA within the 50 μL reaction is digested by 5 U mono-restriction endonuclease at 37 °C/30 min.

Attention ① We found that the selection of the suitable mono-restriction endonuclease should consider the following aspects: (1) it is necessary to consider a relative balance between the number and length of enzyme digestion products; (2) screened endonuclease stability (no easy inactivation); (3) routine (ease of procurement) should also be considered. ② The in-situ substitute/ligation protocol in this experiment has a high ligation efficiency for vector/insert recombinant DNA, while ensuring the integrity of the cpDNA sequence within the length of 90.0 kb.

(Optional) If the ends of the fragments are cohesive, with no blunt end, they are not well-suited for the construction of vector/insert recombinant DNA. Adding 10 U Klenow Fragment into the enzymatic digestion reaction.

#### In-situ substitute

After the MCE membrane was dealt with cold radiation sterilization, the 50 μL enzyme-digested reaction was dripping onto the MCE membrane. It filled a Petri dish with 50 mL of X solution and covered it with a black shell to shield it from light (Fig. 4b), ensuring that the MCE membrane stays floating in the X solution throughout the substitution of the in-situ phase. Using a magnetic stirrer with 0.6 mm spinners, replace the enzyme-digested buffer with the X solution for 8 h at 100 R/4 °C.

Attention ① The X solution is an osmotic alternative for the enzyme-digested buffer and can be osmotically attained for 14–16 μL upon the MEC membrane, that to be the solvent of the clonable cpDNA fragment. The X solution to be the solvent of the clonable cpDNA fragment does not reduce the electroligase efficiency. ② The freshly good-quality cpDNA fragment has a more significant recombinant clone percentage and higher transformation efficiency of the vector-cpDNA fragment in the test. ③ Keeping the processes sterile. ④ The use of phenol for cpDNA extraction is not recommended. We have failed to prepare the high-quality clonable cpDNA fragment several times because of the use of phenol.

#### In-situ ligation

**Fig. 3:**
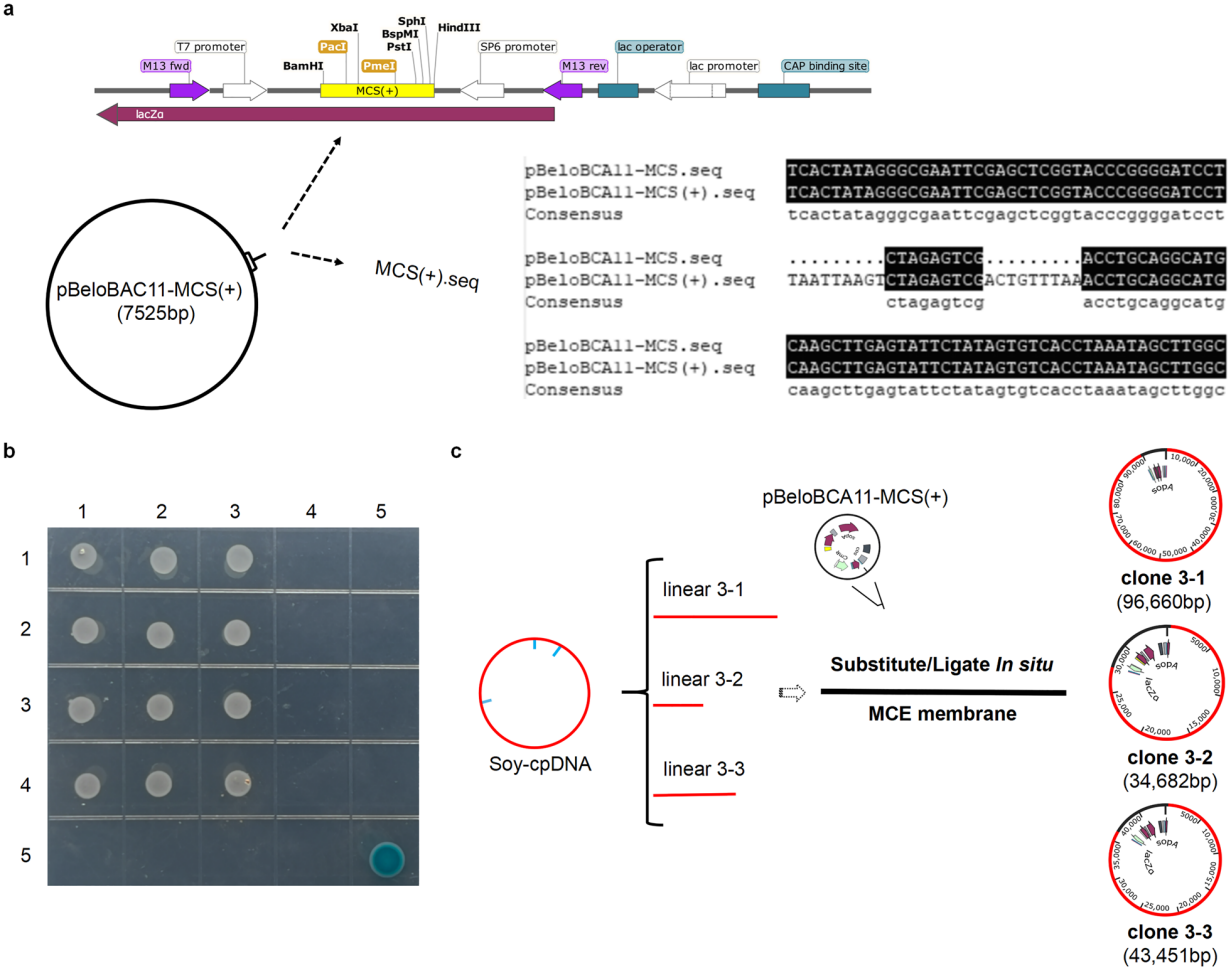
The library of circular cpDNA. **a** The modified plasmid pBeloBAC11-MCS(+). The modified plasmid pBeloBAC11-MCS (+) has been inserted 18 bp on the MCS of pBeloBAC11, adding two standard enzyme-digested sites PacI, PmeI that do not change the blue/white screening. **b** Assembly of the cpDNA libraries on a plate. Conducting the correct colonies from the selective mediums for the cpDNA library construction. A monolithically respectable BAC library is constructed with the circular cpDNA from the crops, marked Wheat by label 1 as 1-1, 1-2, 1-3; marked Maize by label 2, as 2-1, 2-2, 2-3; marked Soybean by label 3, as 3-1, 3-2, 3-3; marked Barley by label 4, as 4-1, 4-2, 4-3. The colony (Label 5-5) shows the blue after transforming the modified plasmid pBeloBAC11-MCS(+) into *E. coil* (DH10B). **c** Illustrations of the soybean circular cpDNA library. Cloneable soybean cpDNA of a suitable fragment inserted into the modifying vector for the formation of vector/insert recombinant DNA

**Fig. 4:**
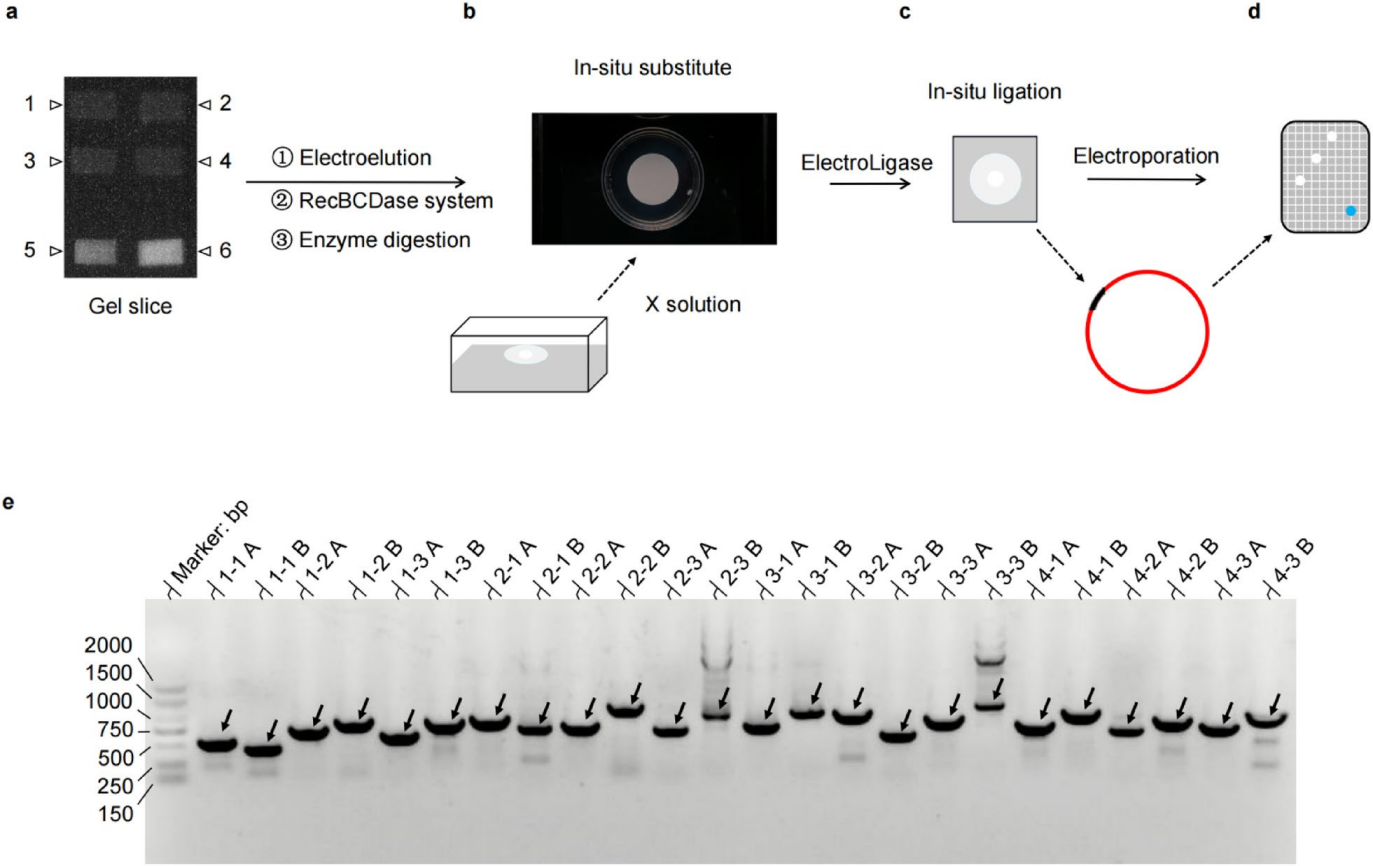
An overview of the procedure for constructing the goal-insert, ordered cpDNA library. **a** Enzyme digestion prepares clonable cpDNA fragments of the desired size. The circular cpDNA within the unstained gel slices represents Wheat (Label 1), Maize (Label 2), Soybean (Label 3) and Barley (Label 4). The gel slices stained with ethidium bromide are Soybean circular cpDNA (label 5, 6), as the +ck of the circular cpDNA template in the slice. The UV then processes the gel slices into the image. **b** In-situ substitute for the desired size, cloneable DNA fragments. The enzyme-digested buffer has been substituted and concentrated by X solution using the MCE membrane as the substrate layer. The MCE membrane was dealt with cold radiation sterilization, and drip the 50 μL enzyme-digested reaction onto the MCE membrane. Using 0.6 mm spinners magnetic stirrer at 100 R/4 °C for 8 h, replace the enzyme-digested buffer with the X solution, and the X solution can be osmotically attained for 14–16 μL upon MEC membrane as the solvent of the clonable cpDNA fragment. **c** In-situ ligation for the good-quality vector/insert recombinant DNA. **d** Construction of the circular cpDNA library. **e** Verifying the cpDNA library by PCR. The colonies from the cpDNA libraries plate are verified by PCR. Black arrowheads show the target bands of the primer sets. The junction sites were validated by PCR, with one PCR primer on the vector and the other on the insert that complies with Table 4. The DL2000 Marker is placed on the far left

Carefully cut the leading edge of the X solution’s osmotic trail within the MCE membrane and insert the MCE slice into the bottom of 2.0 mL RNase-free Microfuge Tubes. To set up ~ 30 μL of ligation reactions, drip the ready-to-use 2× reaction buffer, 0.1% (V/V) PEG 400, linearized vector (cpDNA fragment: vector = 5–10 ng: 1 ng), and electro-ligase (1 μL) into the MCE-slice’s X solution (Fig. 4c). Gentle remixes, as pipetting up and down 7–10 times using the 10 μL wide-bore pipette tip, and incubated with BIOER for 16 °C/8 h. The vector/insert recombinant DNAs of circular cpDNA from four crops are illustrated in Fig. 3c and Additional file 1: Figs. S7–S9.

Chilling the Microfuge Tubes of the ligation reaction on ice for electrotransformation.

Attention ① Storage at − 20 °C is not advised. ② Ligation buffer with PEG 400 consistently produces better results than no PEG 400.

#### Electroporation of vector/insert recombinant DNA

Transform the ligate into *E. coil* (DH10B) by electrotransformation. A respectable amount of recombinant clones per culture dish is achieved (Fig. 4d; Additional file 1: Fig. S2d) by following the instrument operating instructions.

#### Assembly of cpDNA libraries in plate

The positive colonies on selective mediums can be validated by PCR targeting each junction site (with one PCR primer on the vector and the other on the insert; Table 4) and sequencing. Picking the positive colonies for colony PCR validation, the PCR-produced fragments are measured by agarose electrophoresis (Fig. 4e), and the target band is further validated by sequencing. Subsequently, the cpDNA library is constructed using the correct colonies from the selective mediums (Fig. 3b).

**Table 4:** Primer sets for verifying the BCA libraries

Colony	Primers Seq (5′–3′)		
	F:	R:	PCR product (bp)
	GTAAAACGACGGCCAGT (vector)	CAGGAAACAGCTATGAC (vector)	
1-1	TTAAGGACACAAGGTGA	TCTTTATTTCACAAGCGGG	1-1 A (vector F + 1-1 R)
			1-1 B (1-1 F + vector R)
1-2	CCTTGGAACCACCTACAGCT	ACCGTCGGATCACTAAGGC	1-2 A (vector F + 1-2 R)
			1-2 B (1-2 F + vector R)
1-3	GGGAGGAGATGTAAGAAAAATTACCAA	TCGTCCACGGAGGGTGAG	1-3 A (vector F + 1-3 R)
			1-3 B (1-3 F + vector R)
2-1	CGCGAGGGTGAGCTAAC	GGGTTGATCCTAAAGAGATACC	2-1 A (vector F + 2-1 R)
			2-1 B (2-1 F + vector R)
2-2	GCTATATCCCCCCCGAG	CGGGGGCGCTCGTAGTG	2-2 A (vector F + 2-2 R)
			2-2 B (2-2 F + vector R)
2-3	CCATAATGCCTTTCAAATCCTCC	CTCGCCTGCATGAAGCAG	2-3 A (vector F + 2-3 R)
			2-3 B (2-3 F + vector R)
3-1	GAGTTGCTCTTTGGAGAGCAC	AATTGGCGTGCTTGAGG	3-1 A (vector F + 3-1 R)
			3-1 B (3-1 F + vector R)
3-2	CTTCTAGGCAAACCTCCTGGT	AAGCAGGGGCACCTTGC	3-2 A (vector F + 3-2 R)
			3-2 B (3-2 F + vector R)
3-3	CCATCTTTTCTTCAACTTGG	CGGATGTCAGCGGTTCG	3-3 A (vector F + 3-3 R)
			3-3 B (3-3 F + vector R)
4-1	CGAGTATTTGAATACAGCGC	CAAACCAGAACCAGGCGG	4-1 A (vector F + 4-1 R)
			4-1 B (4-1 F + vector R)
4-2	CTGACACAGCTTCTGAATG	GAAAGACAATTTGTCGCAC	4-2 A (vector F + 4-2 R)
			4-2 B (4-2 F + vector R)
4-3	CGCCATTTCAAACCTGA	TCCCAATAGATGTGACCGA	4-3 A (vector F + 4-3 R)
			4-4 B (4-3 F + vector R)

The PCR to target each of the junction site with one PCR primer on the vector and the other on the insert

### Verifying the cpDNA library quality

#### Using PCR and sequencing to verify the cpDNA library quality

PCR for ascertaining the junction sites of vector/insert recombinant DNA from the libraries (Fig. 4e).

#### cpDNA library sequencing and gene annotation

The genomic library of soybean cpDNA library was prepared for sequencing on Illumina platforms with PE150 strategy, following the manufacturer’s recommended protocol (Illumina, California, USA). After removal of DH10B genome sequences. The DNA library generated 3.25 Gb clean data and the average sequencing depth was 21,348.70x. The sequencing data was of high quality (Q 20 = 96.8%, Q 30 = 91.5%).

Bowtie2 v2.5.1 (https://bowtie-bio.sourceforge.net/bowtie2/index.shtml), Samtools v1.18 (http://www.htslib.org/), SPAdes v3.13.0 (https://cab.spbu.ru/software/spades/), A5-miseq v20160825 (https://sourceforge.net/projects/ngopt/files/), and Gapfiller v2.1.2 (https://sourceforge.net/projects/gapfiller/files/) assembly software were used separately to assemble the clean data. The primary assembly yielded a complete circular cpDNA of soybean. After annotation of regions by alignment against the soybean chloroplast Ref-genome (NC_007942) by the online software CPGAVAS2 (http://47.96.249.172:16019/analyzer/annotate), oligonucleotide primers were designed to amplify across problematic, remaining gap regions, and polymorphism site.

## Results

### In-situ chloroplast lysis

A novel circular cpDNA enrichment approach incorporating a set of original buffers/solutions and optimized steps is suitable for the crop’s circular cpDNA extraction (Fig. 2). These procedures are different from the bacterium’s peptidoglycan/mucopeptide lysis in situ. It is the chief cause of the circular cpDNA, without breakage, presenting a distinct optimal concentration in the gel (Fig. 2b). Meanwhile, the MES and NaCl stabilize the microenvironment in circular cpDNA to a pH of no more than 8.0 and a sufficient concentration of salt ions.

In the crude chloroplast preparation step, the original ET-M-D buffer immerses the leaves for 10–20 min at 4 °C to reduce the cpDNA degradation (Fig. 2a1). In the in-gel chloroplast lysis step, the original Et-S buffer incubates the in-gel at 35 °C/2.5 h helping to increase the cpDNA release (Fig. 2a2). Comparison of the lysing abilities of the Et-S buffer for in-gel chloroplast by a temporal gradient, showing in an autoradiogram from 1% PFGE-gel analysis (Fig. 2c). The naked cpDNA within gelled agarose (in-gel cpDNA; Fig. 2a3) is enriched and purified at the genomic level by PFGE for up to 16 h.

The circular cpDNA in the unstained gel slice is extracted by Electroelution. Releasing the circular cpDNA from the unstained gel slices (Additional file 1: Fig. S2a) using the Dialysis tube, meanwhile, the 5 mM Tris-HCl (pH = 8.0) as the running buffer (Fig. 2a4; Additional file 1: Fig. S2b) and then apply 90 V electric current for 45 min until the circular cpDNA exited the unstained gel slice.

The linear nucleic acid impurities of circular cpDNA are digested by the RecBCDase system. RecBCDase system (Table 3) is crucial in cleaning the non-target impurities within circular cpDNA solution. The 2 U RecBCDase system with 3 mM ATPs at 37 °C/10 h and 70 °C/10 min usually yields tens of nanograms of highly pure circular cpDNA.

In general, this newly circular cpDNA extraction method is appropriate for circular cpDNA extraction from the crops (Fig. 2d).

### In-situ substitute/ligation

The in-situ substitute/ligation method takes a high ligation efficiency of the vector/insert recombinant DNA while ensuring the integrity of the cpDNA sequence within 90 kb. which is necessary to construct the goal-insert, ordered cpDNA library (Fig. 3b) to replace the conventional, unordered ones. One cpDNA library includes the circular cpDNA of one crop by three colonies with the cpDNA fragments of the desired size (Fig. 3c).

A modified plasmid pBeloBAC11-MCS (+) has been demonstrated in our lab (Fig. 3a). Two standard enzyme-digested sites, PacI and PmeI, that do not alter the blue/white screening (Fig. 3b5) have been inserted into the MCS of plasmid pBeloBAC11, which help for the effective ligation of the blunt-end of the kilobase-sized DNA.

High-quality cpDNA is crucial to constructing the goal-insert, ordered cpDNA library. The kilobase-sized cpDNAs from the mono-restriction endonuclease after the in-situ chloroplast lysis procedure are prepared for use (Additional file 1: Fig. S2c). Generating clonable fragments of the correct size is a complex technique comprising multiple steps: the mono-restriction endonuclease digestion, repairing the enzyme-digested products by end-blunting (Fig. 4a), and in-situ substitute with X solution. The X solution (which does not affect the ligation efficiency) will substitute the enzyme-digested buffer by in-situ substitute protocol and does not need a manual operation that avoids the external forces (Fig. 4b).

Several factors are crucial to ensuring the efficient ligation to construct the vector/insert recombinant DNA. The goal-clonable cpDNAs (Additional file 1: Fig. S2c) are prepared by the in-situ substitute protocol (Fig. 4b with X solution) after the circular cpDNA has been digested by the mono-restriction endonuclease. The vector/insert recombinant DNA is ligated by the in-situ ligation protocol (Fig. 4c). Electroporation of vector/insert recombinant DNA (Fig. 4d) and assembly of cpDNA libraries in the plate (Fig. 3b).

Furthermore, the in-situ substitute/ligation protocol can not be limited to making the goal-clonable cpDNAs, it also used for the other nucleic acid sequences to produce the kilobase-sized, goal-clonable DNA fragments and construct the goal-insert, ordered DNA library.

### Verifying the quality of the cpDNA library

PCR for verifying the junction sites of vector/insert recombinant DNA from the cpDNA libraries (Fig. 4e). The colonies from the plate of the cpDNA library (Fig. 3b) are verified by PCR, with one PCR primer on the vector and the other on the insert (Table 4), the target bands marked by black arrowheads.

**Fig. 5:**
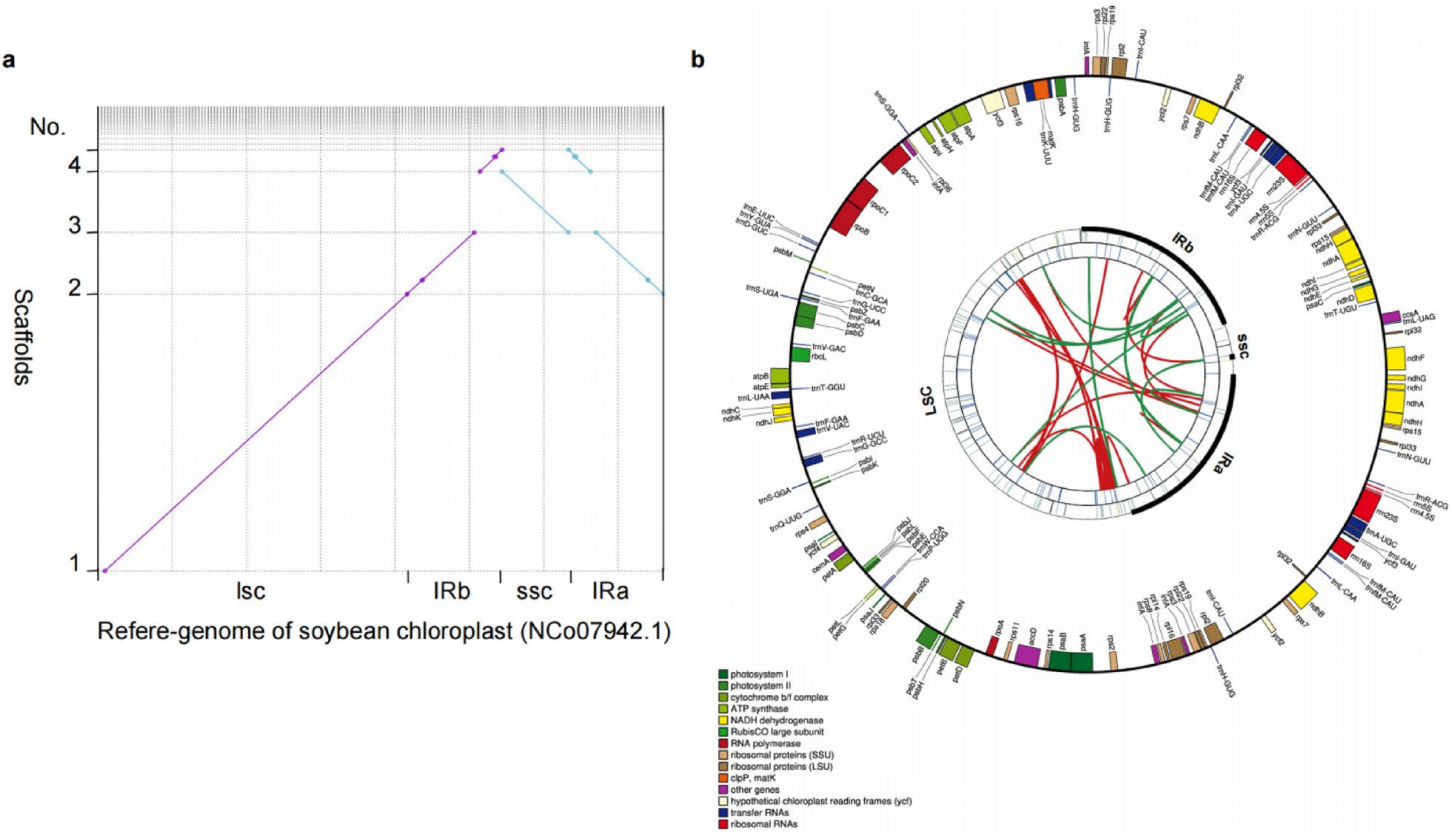
Verifying the quality of the cpDNA library. **a** Verify the cpDNA library’s quality using the sequencing results. The scaffold fasta file is constructed from the sequencing result by SPAdes and A5-miseq software. Collinearity analysis to verify the clones of the cpDNA sequence integrity from soybean circular cpDNA library (conform to Fig. 2b3). The reference genome of soybean chloroplast (NCo 07942.1) is entirely covered with the four scaffolds by the MUMmer software. Only five gaps/insertions with an average size of 21.6 bp were visible and located at specific points in the Referee-genome’s IRa and IRb. LSC and SSC refer to the large and small single-copy regions, respectively, and IRa and IRb are the two inverted repeats. **b** Diagrammatic representation of the circular cpDNA traits in soybeans. Gap-filling by PCR was performed after screening/assembling the four scaffolds from the scaffold fasta file. The CPGAVAS2 software can annotate and view the soybean circular cpDNA from the cpDNA libraries. From the center going outward, the first circle shows the forward and reverse repeats connected with red and green arcs, respectively. The second circle shows the tandem repeats marked with short bars. The third circle shows the microsatellite sequences identified using MISA. The fourth circle is drawn using draw gene map and shows the gene structure on the soybean circular cpDNA. The genes are colored based on their functional categories

Following sequencing, the scaffold fasta file is assembled from the sequencing results by the SPAdes and A5-miseq software. Collinearity analysis for verifying the clones of the cpDNA sequence integrity from the circular cpDNA library of Soybean (Fig. 3b3). Four scaffolds (no. 1, 2, 3, and 4) from the scaffold fasta file are assembled and gap-filling by sequencing the PCR. The reference genome of soybean chloroplast is entirely covered with the four scaffolds by the MUMmer software (Fig. 5a). Only five gaps/insertions with an average size of 21.6 bp are visible, and they are situated at specific points in the Refere-genome’s IRa and IRb (Fig. 5a2, 4).

The assembled result of soybean circular genome from the cpDNA library of Soybean was annotated/viewed by the CPGAVAS2 software. LSC and SSC refer to the large and small single-copy regions, respectively, and IRa and IRb are the two inverted repeats. From the center going outward, the first circle shows the forward and reverse repeats connected with red and green arcs respectively. The second circle shows the tandem repeats marked with short bars. The third circle shows the microsatellite sequences identified using MISA. The fourth circle is drawn using drawgenemap and shows the gene structure on the soybean circular cpDNA. The gene is colored based on their functional categories (Fig. 5b).

## Discussion

Technological innovation is probably the most important catalyst for progress in any scientific discipline. In crops with large nuclear genomes, homology between nuclear and organellar genomes could interfere with assembly and analysis. Enriching (purest and high-yield) circular cpDNA to reduce sequence complexity for cpDNA research is advantageous.

### In-situ-process

After the in-situ chloroplast lysis used for cp genome structural research in 1989 [3], it has not been used for the cpDNA extraction field till now. In the meantime, the reagents and the steps which are unaltered quoted from the in-situ bacterial lysis method, are inefficient in chloroplast lysis (Additional file 1: Fig. S10), because of the difference between the peptidoglycan/mucopeptide of the bacterial cell wall and lipid bilayer of chloroplast membrane.

For whole circular cpDNA extraction, the conventional extraction method with an aqueous phase (like the CTBA method and alkaline lysis) caused the fragmentation of the circular cpDNA (Additional file 1: Fig. S11) and a few residual non-target DNA impurities are inevitable [45, 47]. Yet the advantageous factors are undeniable in conventional extraction methods, like a relatively simple process and time cost.

Based on the result as described above, for the high-purity circular cpDNA from chloroplast, we learn from its principle of the in-situ technique for bacterial lysis that could keep the DNA non-breakage and optimize the lysis system. A protocol of in-situ chloroplast lysis for the circular cpDNA has been developed with a series of original buffers/solutions and optimized procedures, that lyse the chloroplast within gelled agarose and keep the chloroplast genome non-breakage. The circular cpDNA within gelled agarose will be enriched and purified at the genomic level by PFGE for up to 16 h. This technique is very simple in operation (does not need centrifuge, mix uniform, and so on) except during the chloroplast embedding and has a high resultant repetition rate for circular cpDNA extraction.

Specific steps and the theories are elaborated as follows: ① The ET-M-D buffer is used for inactivating the cell protease, and then the crude chloroplasts are isolated by centrifugation following the inactivation of the cell protease. The E-T-MD buffer used for macerate the leaves before grinding them, in order to keep the dynamic equilibrium of intracellular and interstitial fluids by the organic solute and d-mannitol, that is necessary for the organic solute to inactivate the cell proteases (preventing cpDNA degradation). After the organic solute enters cells by simple diffusion, d-mannitol works to offset the consequences of the high intracellular pressure, protecting the integrity of the cell membrane system; ② The Et-S buffer is used for chloroplast lysis in-situ. The lipid bilayer of chloroplast membrane is lysed, and the chlorophyll and chloroplast’s inner protoplasm are separated out the gelled agarose. It is plausible to infer that the in-situ-process technique could be applied also to the other organelles.

The electrochemical method that giving a stable low-voltage condition to electroelution, to ensure the integrity of the whole circular cpDNA. The linear nucleic acid impurities of cpDNA are digested by the RecBCDase system. Thus, high-purity whole circular cpDNA has been achieved.

### In-situ substitute/ligation

We have successfully constructed the goal-insert, ordered cpDNA library to replace the conventional, unordered ones, that the cpDNA fragment is random from the conventional extraction method. Which can successfully preserve the whole circular cpDNA in three colonies.

Good-quality vector/insert recombinant DNA produced by the in-situ substitute/ligation method is crucial to a successful cpDNA library. Creating clonable fragments of the desired size involves a multi-step process: ① keeping the enzyme-digested cpDNA on the MCE membrane and replacing the enzyme-digested buffer. The X solution (which does not affect the ligation efficiency) will take the place of the enzyme-digested buffer by in-situ substitute protocol and does not need the manual operation that avoids the external forces (like multiple-centrifuge, multiple-mixes uniform), and keeps the integrity of the cpDNA sequence that within the length of 90.0 kb; ② ligating in situ for vector/insert recombinant DNA while ensuring the integrity of the cpDNA sequence. The targeted ligation of vector/insert recombinant DNA by the in-situ ligation protocol which dripping the ligation buffer and the linearized vector into the MCE-slice’s X solution, could minimize the external forces influence as much as possible, which will effectively improve the targeted ligation efficiency.

This procedure continues to be challenging and highly skill- and bench-experience-demanding. Therefore, a few attempts are likely needed for a researcher with less research experience with the technique to construct a good-quality cpDNA library.

## Conclusions

In-situ chloroplast lysis is developed to lyse the chloroplast within gelled agarose and keep the cp genome non-breakage. The circular cpDNA at the genomic level will be enriched and purified within gelled agarose by PFGE method, and the whole circular cpDNA is isolated by the electrochemical method that gives a stable low-voltage condition in electroelution, to ensure the integrity of the whole circular cpDNA. The in-situ substitute/ligation protocol is developed to construct the goal-insert, ordered cpDNA library, that can stably insert the target cpDNA sequence within 90.0 kb. Which can successfully preserve the whole circular cpDNA in three colonies. In addition to constructing the goal-insert, ordered cpDNA library, in-situ substitute/ligation protocol can used for the other nucleic acid sequence with a ten-digit kb level to produce the large-clonable DNA fragments and construct the goal-insert DNA libraries.

In short, this study developed a set of high-purity circular cpDNA extraction and goal-insert, ordered cpDNA library construction methods. At the same time, can be used to construct the large-insert, ordered DNA library, that is amply for further experiments, such as genomic sequencing, bioinformatics analyses, cloning, and physical mapping.

### Supplementary Information

**Additional file 1: Figure S1.** (a) Sample of crop leaves. Using Hydroponics with the repeated-short time scale of lighting, reduced the carbohydrate content of the leaves. Respectively, the labels (1 to 4) are Barley (3–4 leaf stage), Wheat (3–4 leaf stage), Maize (V2–V3 stage), and Soybean (V3–V4 stage). The yellow and white arrowheads represent younger (V2 stage) and older (V6 stage) age groups. The soybean older leaves (white line), before and after the V6 stage, the leaves have turned yellow and entered the leaf senescence development. Whereas, under natural conditions, the V6 stage of soybean is at the beginning of vegetative growth. (b) Chloroplast images. Soybean cell and chloroplast are distinguished by labels 1 and 2, respectively. The black arrowheads denote the cell, and the red arrowheads denote the chloroplast. Left bar = 20 μm, right bar = 50 μm. **Figure S2.** (a) Circular cpDNA extraction. The remaining gel (Lane 1, 2 and 3, red arrowhead) was stained with ethidium bromide before reconstructing the integrated gel to cut the elutable gel slices that contained the unstained circular cpDNA (Lane 4, 5 and 6). Subjecting to PFGE using the following procedure: Reverse Voltage Gradient: 4.5 V/cm, Int. Sw. Tm: 60 s, Fin. Sw. Tm:90 s, Included angle: 120°, Run Time: 18 h, Ramping factor: a=: ENTER. The HMW lambda ladder loaded into the far left. Attention circular DNA size cannot be marked up with a linearized DNA ladder because of the circular topology properties, whereas it can serve as a relative reference standard. (b) Recovering the circular cpDNA from gel slices using the D-tube Dialyzer. A supporting tray containing the unstained gel slice is built by placing the unstained gel slice into the D-Tube dialyzer. Filling the D-tube dialyzer with 5 mM Tris-HCl running buffer to the top of membranes. Avoid introducing air bubbles in the tube, as they interfere with electroelution. The optimum electroelution time must be determined according to the sample and gel concentration. (c) The mono-restriction endonuclease. SrfI, BssHII, NaeI and SacII are screened to digest the circular cpDNA of Wheat (Label 1), Maize (Label 2), Soybean (Label 3) and Barley (Label 4), respectively. End-blunting the cpDNA fragments by the Klenow Fragment when the end of the fragments is a cohesive end. The redly label numbers are marked with the enzyme-digested products. (d) Electroporation of vector/insert recombinant DNA. Transform the ligate into *E. coil* (DH10B) by electrotransformation. A respectable amount of recombinant clones per culture dish is achieved with the circular cpDNA from Wheat (Label 1), Maize (Label 2), Soybean (Label 3) and Barley (Label 4), respectively. **Figure S3.** Illustration of the mono-restriction endonuclease selection from Wheat chloroplast Ref-genome. **Figure S4.** Illustration of the mono-restriction endonuclease selection from Maize chloroplast Ref-genome. **Figure S5.** Illustration of the mono-restriction endonuclease selection from Soybean chloroplast Ref-genome. **Figure S6.** Illustration of the mono-restriction endonuclease selection from Barley chloroplast Ref-genome. **Figure S7.** Illustration of the wheat circular cpDNA library. To create a vector/insert recombinant DNA, a cloneable wheat cpDNA fragment of the desired size is introduced into the modifying vector. **Figure S8.** Illustration of the maize circular cpDNA library. To create a vector/insert recombinant DNA, a cloneable maize cpDNA fragment of the desired size is introduced into the modifying vector. **Figure S9.** Illustrations of the barley circular cpDNA library. To create a vector/insert recombinant DNA, a cloneable barley cpDNA fragment of the desired size is introduced into the modifying vector. **Figure S10.** Lysate efficacy of the old lysate in the In-gel chloroplast. The reagents and the steps, which are unaltered and quoted from the in-situ bacterial lysis method, are used to lyse the In-gel chloroplast (soybean). Labels 1, 2, and 3 are three repetitions. Subjecting to PFGE using the following procedure: Reverse Voltage Gradient: 6 V/cm, Int. Sw. Tm: 60 s, Fin. Sw. Tm: 90 s, included angle: 120°, Run Time: 18 h, Ramping factor: a=: ENTER. The HMW lambda ladder loaded into the far left. **Figure S11.** Comparison of whole circular cpDNA extraction methods conventional technique and in-situ-process technique. (a) Discontinuous sucrose gradient is prepared for chloroplast purification. The upper layer of Label 1 is soybean crude chloroplast, and the lower layer of Label 1 is discontinuous sucrose density gradients (8%, 25%, 45%, 60%, and 80%). (b) Purification of crude chloroplast. The intact chloroplasts approach the equilibrium position in the lower band (Label 3), whereas non-target pollutants in the label 1 and damaged chloroplat in the label 2. Label 1 is the larger non-target pollutants like surviving leaves tissue, intact cells, and other debris. Centrifugal condition: 10,000×*g*/60 min/4 °C. (c) Detecting the extracting effect of circular cpDNA by PFGE-gel. The soybean circular cpDNA from the in-situ chloroplast lysis technique cannot be separated as discrete bands under PGFE conditions, showing the target band (line 2) in PFGE-gel. Whereas the conventional technique has separated as discrete bands (line 1) under PGFE. PFGE conditions: Reverse Voltage Gradient: 6 V/cm, Int. Sw. Tm: 1 s, Fin. Sw. Tm: 20 s, included angle: 120°, Run Time: 14 h, Ramping factor: a=: ENTER). line NA, not available. The HMW lambda ladders are loaded into the first and second on the left corner.

**Additional file 2: Table S1.** A summary of the basic components and procedures for optimizing chloroplast and cpDNA extraction.

## Data Availability

All the data generated or analyzed during this study are included within this article.

## Abbreviations

In-situ-process: Combines in-situ chloroplast lysis with in-situ substitute/ligation
PFGE: Pulse field gel electrophoresis
Mb: Mega-base pair
Kb: Kilo-base pair
cp genome: Chloroplast genome
cpDNA: Chloroplast DNA
