# Supplementary figures and images for: A novel in-situ-process technique constructs whole circular cpDNA library
Source: Plant Methods. 2024 Jan 3;20:2. doi: 10.1186/s13007-023-01126-7 (PMC10763311; doi:10.1186/s13007-023-01126-7)

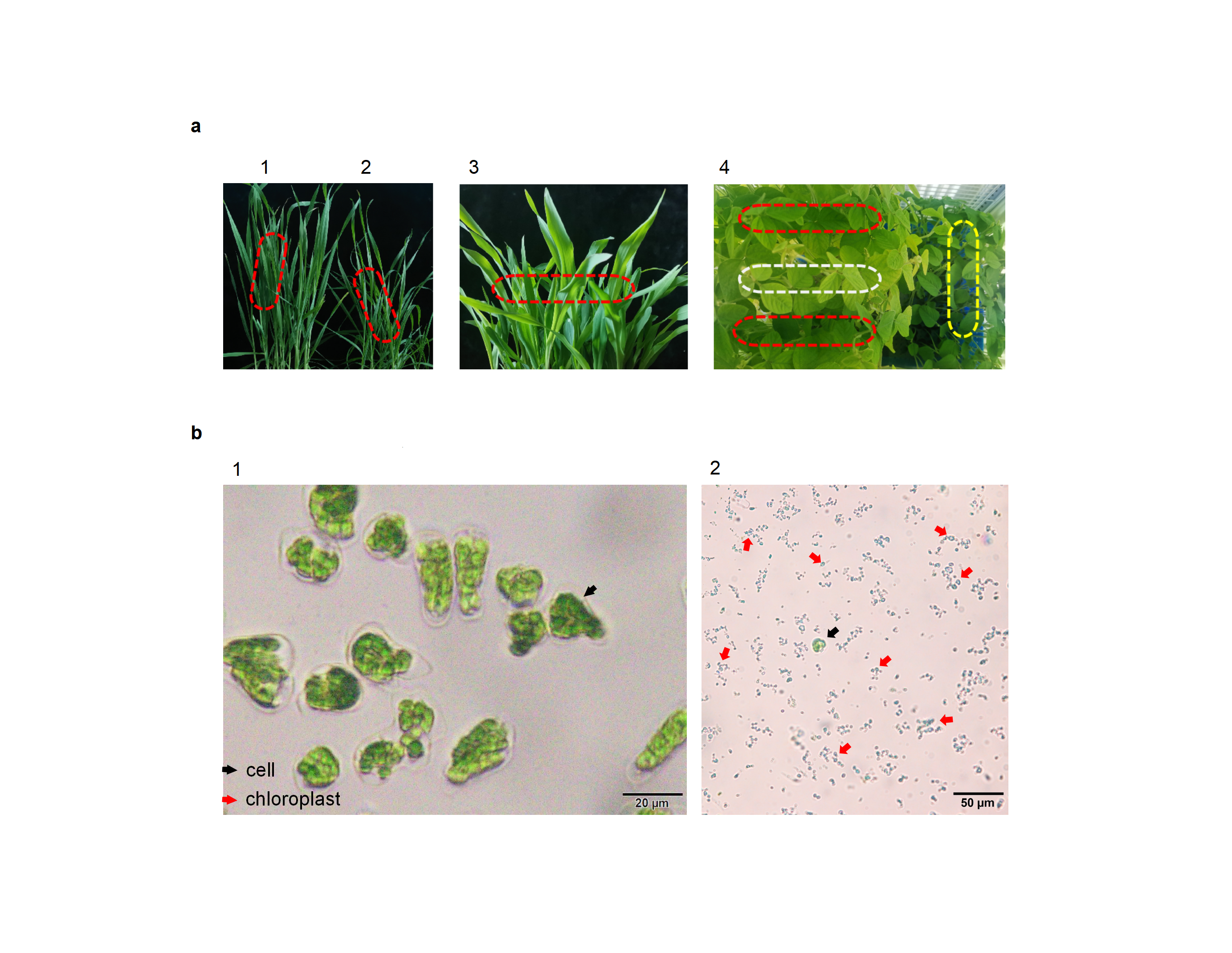

Supplement: Supplementary file 1 — Additional file 1: Figure S1. (a) Sample of crop leaves. Using Hydroponics with the repeated-short time scale of lighting, reduced the carbohydrate content of the leaves. Respectively, the labels (1 to 4) are Barley (3–4 leaf stage), Wheat (3–4 leaf stage), Maize (V2–V3 stage), and Soybean (V3–V4 stage). The yellow and white arrowheads represent younger (V2 stage) and older (V6 stage) age groups. The soybean older leaves (white line), before and after the V6 stage, the leaves have turned yellow and entered the leaf senescence development. Whereas, under natural conditions, the V6 stage of soybean is at the beginning of vegetative growth. (b) Chloroplast images. Soybean cell and chloroplast are distinguished by labels 1 and 2, respectively. The black arrowheads denote the cell, and the red arrowheads denote the chloroplast. Left bar = 20 μm, right bar = 50 μm. Figure S2. (a) Circular cpDNA extraction. The remaining gel (Lane 1, 2 and 3, red arrowhead) was stained with ethidium bromide before reconstructing the integrated gel to cut the elutable gel slices that contained the unstained circular cpDNA (Lane 4, 5 and 6). Subjecting to PFGE using the following procedure: Reverse Voltage Gradient: 4.5 V/cm, Int. Sw. Tm: 60 s, Fin. Sw. Tm:90 s, Included angle: 120°, Run Time: 18 h, Ramping factor: a=: ENTER. The HMW lambda ladder loaded into the far left. Attention circular DNA size cannot be marked up with a linearized DNA ladder because of the circular topology properties, whereas it can serve as a relative reference standard. (b) Recovering the circular cpDNA from gel slices using the D-tube Dialyzer. A supporting tray containing the unstained gel slice is built by placing the unstained gel slice into the D-Tube dialyzer. Filling the D-tube dialyzer with 5 mM Tris-HCl running buffer to the top of membranes. Avoid introducing air bubbles in the tube, as they interfere with electroelution. The optimum electroelution time must be determined according to the sample and gel c [file 13007_2023_1126_MOESM1_ESM.zip › Additional file 1 Figs - labels/Figure S1.tif]

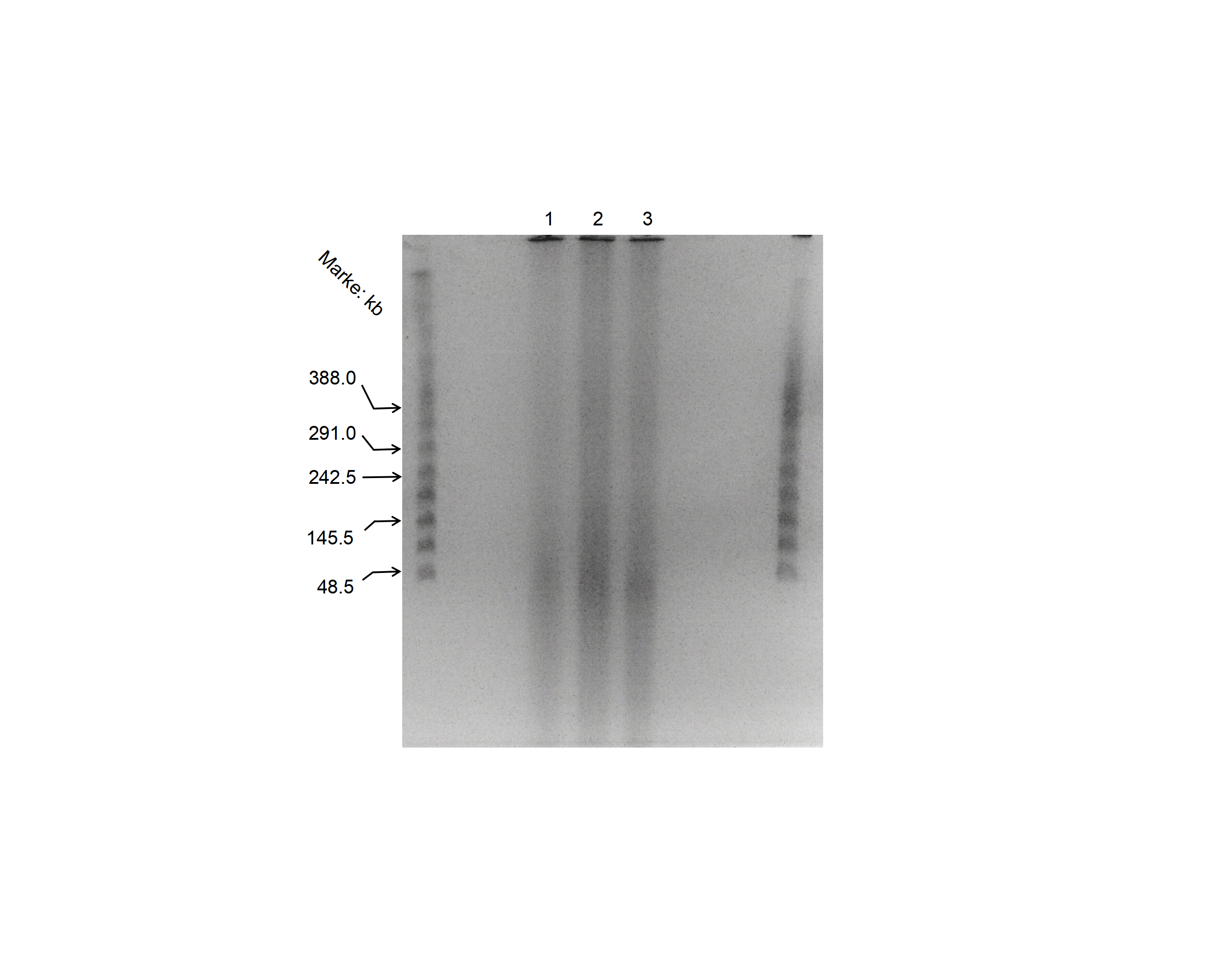

Supplement: Supplementary file 1 — Additional file 1: Figure S1. (a) Sample of crop leaves. Using Hydroponics with the repeated-short time scale of lighting, reduced the carbohydrate content of the leaves. Respectively, the labels (1 to 4) are Barley (3–4 leaf stage), Wheat (3–4 leaf stage), Maize (V2–V3 stage), and Soybean (V3–V4 stage). The yellow and white arrowheads represent younger (V2 stage) and older (V6 stage) age groups. The soybean older leaves (white line), before and after the V6 stage, the leaves have turned yellow and entered the leaf senescence development. Whereas, under natural conditions, the V6 stage of soybean is at the beginning of vegetative growth. (b) Chloroplast images. Soybean cell and chloroplast are distinguished by labels 1 and 2, respectively. The black arrowheads denote the cell, and the red arrowheads denote the chloroplast. Left bar = 20 μm, right bar = 50 μm. Figure S2. (a) Circular cpDNA extraction. The remaining gel (Lane 1, 2 and 3, red arrowhead) was stained with ethidium bromide before reconstructing the integrated gel to cut the elutable gel slices that contained the unstained circular cpDNA (Lane 4, 5 and 6). Subjecting to PFGE using the following procedure: Reverse Voltage Gradient: 4.5 V/cm, Int. Sw. Tm: 60 s, Fin. Sw. Tm:90 s, Included angle: 120°, Run Time: 18 h, Ramping factor: a=: ENTER. The HMW lambda ladder loaded into the far left. Attention circular DNA size cannot be marked up with a linearized DNA ladder because of the circular topology properties, whereas it can serve as a relative reference standard. (b) Recovering the circular cpDNA from gel slices using the D-tube Dialyzer. A supporting tray containing the unstained gel slice is built by placing the unstained gel slice into the D-Tube dialyzer. Filling the D-tube dialyzer with 5 mM Tris-HCl running buffer to the top of membranes. Avoid introducing air bubbles in the tube, as they interfere with electroelution. The optimum electroelution time must be determined according to the sample and gel c [file 13007_2023_1126_MOESM1_ESM.zip › Additional file 1 Figs - labels/Figure S10.tif]

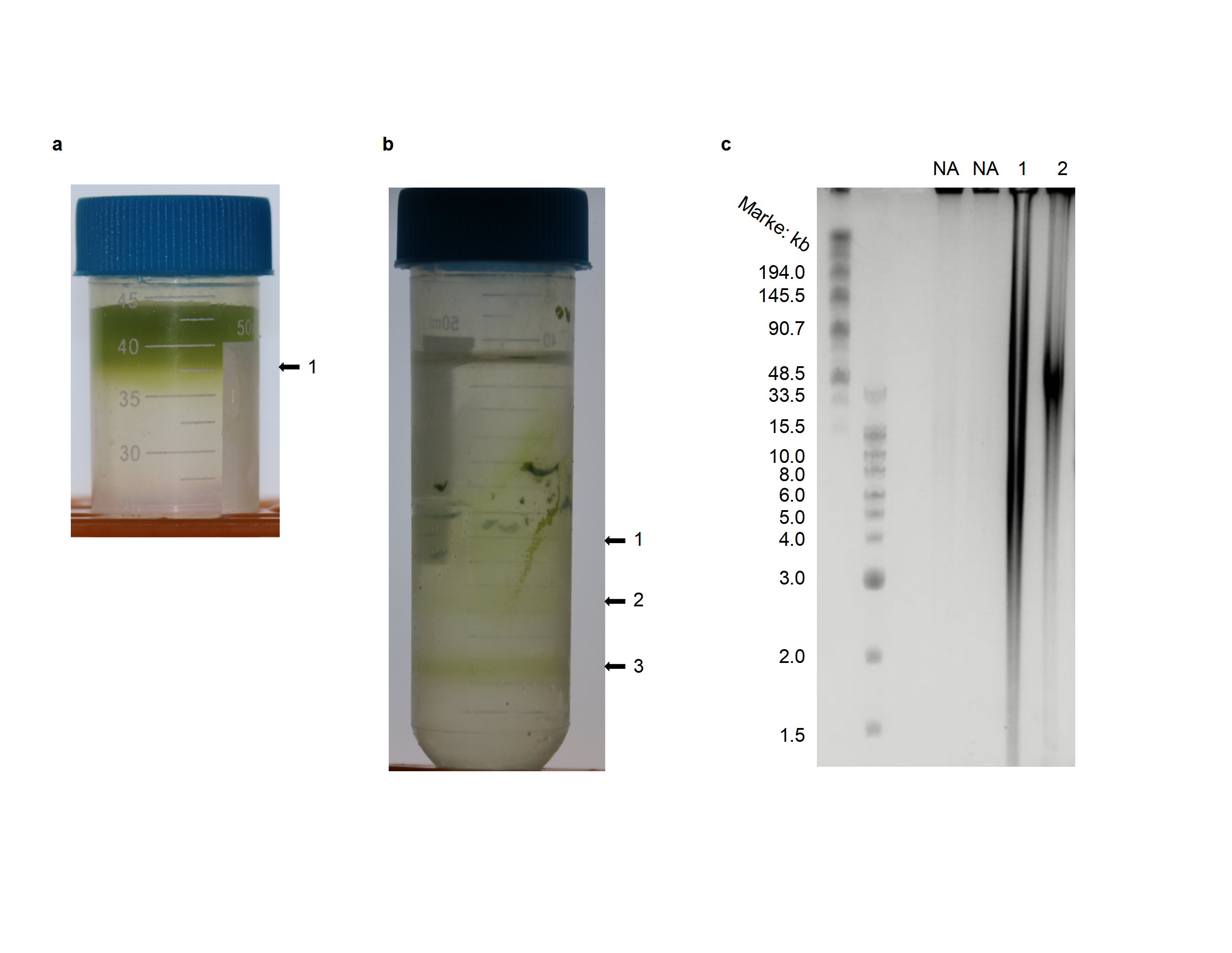

Supplement: Supplementary file 1 — Additional file 1: Figure S1. (a) Sample of crop leaves. Using Hydroponics with the repeated-short time scale of lighting, reduced the carbohydrate content of the leaves. Respectively, the labels (1 to 4) are Barley (3–4 leaf stage), Wheat (3–4 leaf stage), Maize (V2–V3 stage), and Soybean (V3–V4 stage). The yellow and white arrowheads represent younger (V2 stage) and older (V6 stage) age groups. The soybean older leaves (white line), before and after the V6 stage, the leaves have turned yellow and entered the leaf senescence development. Whereas, under natural conditions, the V6 stage of soybean is at the beginning of vegetative growth. (b) Chloroplast images. Soybean cell and chloroplast are distinguished by labels 1 and 2, respectively. The black arrowheads denote the cell, and the red arrowheads denote the chloroplast. Left bar = 20 μm, right bar = 50 μm. Figure S2. (a) Circular cpDNA extraction. The remaining gel (Lane 1, 2 and 3, red arrowhead) was stained with ethidium bromide before reconstructing the integrated gel to cut the elutable gel slices that contained the unstained circular cpDNA (Lane 4, 5 and 6). Subjecting to PFGE using the following procedure: Reverse Voltage Gradient: 4.5 V/cm, Int. Sw. Tm: 60 s, Fin. Sw. Tm:90 s, Included angle: 120°, Run Time: 18 h, Ramping factor: a=: ENTER. The HMW lambda ladder loaded into the far left. Attention circular DNA size cannot be marked up with a linearized DNA ladder because of the circular topology properties, whereas it can serve as a relative reference standard. (b) Recovering the circular cpDNA from gel slices using the D-tube Dialyzer. A supporting tray containing the unstained gel slice is built by placing the unstained gel slice into the D-Tube dialyzer. Filling the D-tube dialyzer with 5 mM Tris-HCl running buffer to the top of membranes. Avoid introducing air bubbles in the tube, as they interfere with electroelution. The optimum electroelution time must be determined according to the sample and gel c [file 13007_2023_1126_MOESM1_ESM.zip › Additional file 1 Figs - labels/Figure S11.tif]

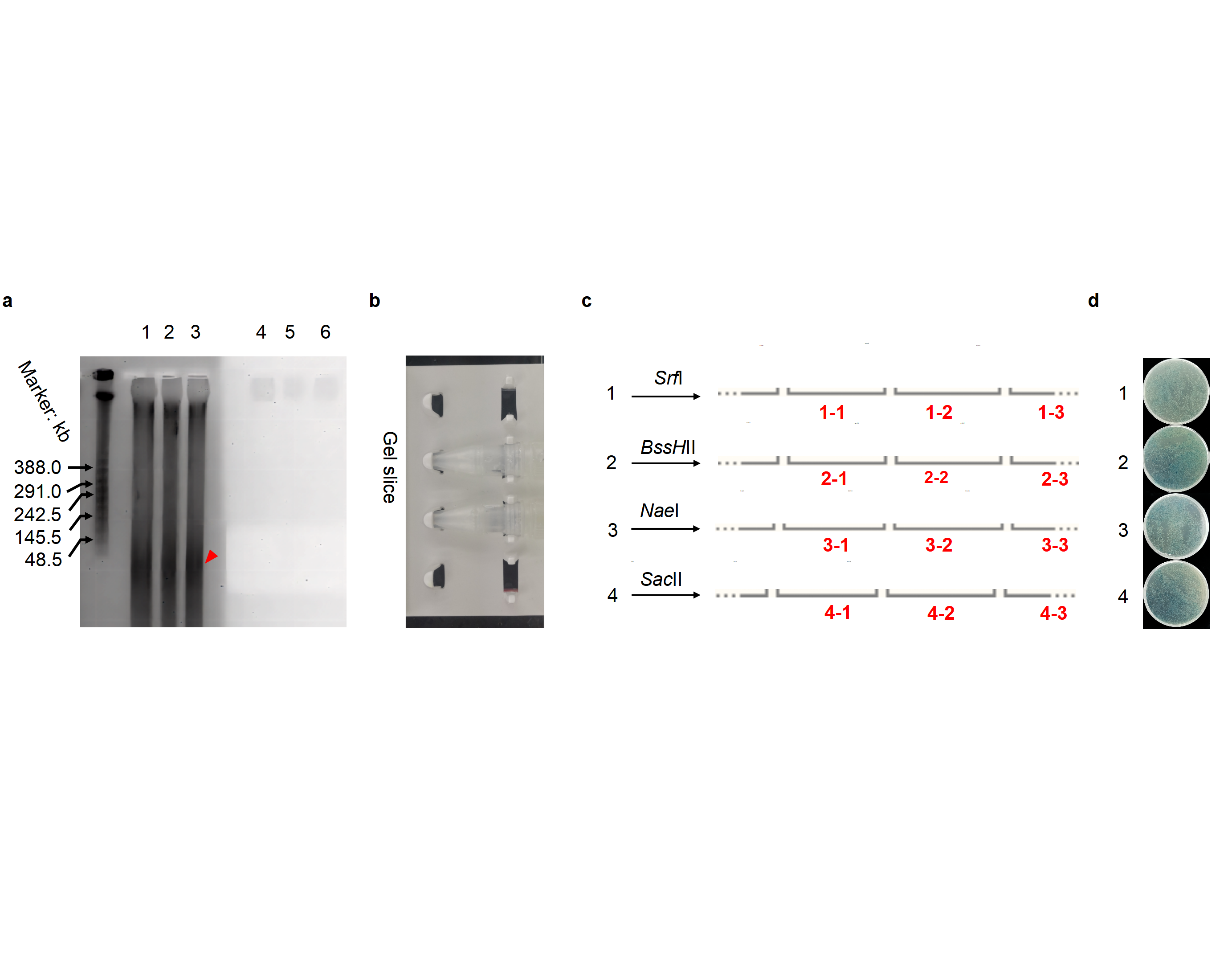

Supplement: Supplementary file 1 — Additional file 1: Figure S1. (a) Sample of crop leaves. Using Hydroponics with the repeated-short time scale of lighting, reduced the carbohydrate content of the leaves. Respectively, the labels (1 to 4) are Barley (3–4 leaf stage), Wheat (3–4 leaf stage), Maize (V2–V3 stage), and Soybean (V3–V4 stage). The yellow and white arrowheads represent younger (V2 stage) and older (V6 stage) age groups. The soybean older leaves (white line), before and after the V6 stage, the leaves have turned yellow and entered the leaf senescence development. Whereas, under natural conditions, the V6 stage of soybean is at the beginning of vegetative growth. (b) Chloroplast images. Soybean cell and chloroplast are distinguished by labels 1 and 2, respectively. The black arrowheads denote the cell, and the red arrowheads denote the chloroplast. Left bar = 20 μm, right bar = 50 μm. Figure S2. (a) Circular cpDNA extraction. The remaining gel (Lane 1, 2 and 3, red arrowhead) was stained with ethidium bromide before reconstructing the integrated gel to cut the elutable gel slices that contained the unstained circular cpDNA (Lane 4, 5 and 6). Subjecting to PFGE using the following procedure: Reverse Voltage Gradient: 4.5 V/cm, Int. Sw. Tm: 60 s, Fin. Sw. Tm:90 s, Included angle: 120°, Run Time: 18 h, Ramping factor: a=: ENTER. The HMW lambda ladder loaded into the far left. Attention circular DNA size cannot be marked up with a linearized DNA ladder because of the circular topology properties, whereas it can serve as a relative reference standard. (b) Recovering the circular cpDNA from gel slices using the D-tube Dialyzer. A supporting tray containing the unstained gel slice is built by placing the unstained gel slice into the D-Tube dialyzer. Filling the D-tube dialyzer with 5 mM Tris-HCl running buffer to the top of membranes. Avoid introducing air bubbles in the tube, as they interfere with electroelution. The optimum electroelution time must be determined according to the sample and gel c [file 13007_2023_1126_MOESM1_ESM.zip › Additional file 1 Figs - labels/Figure S2.tif]

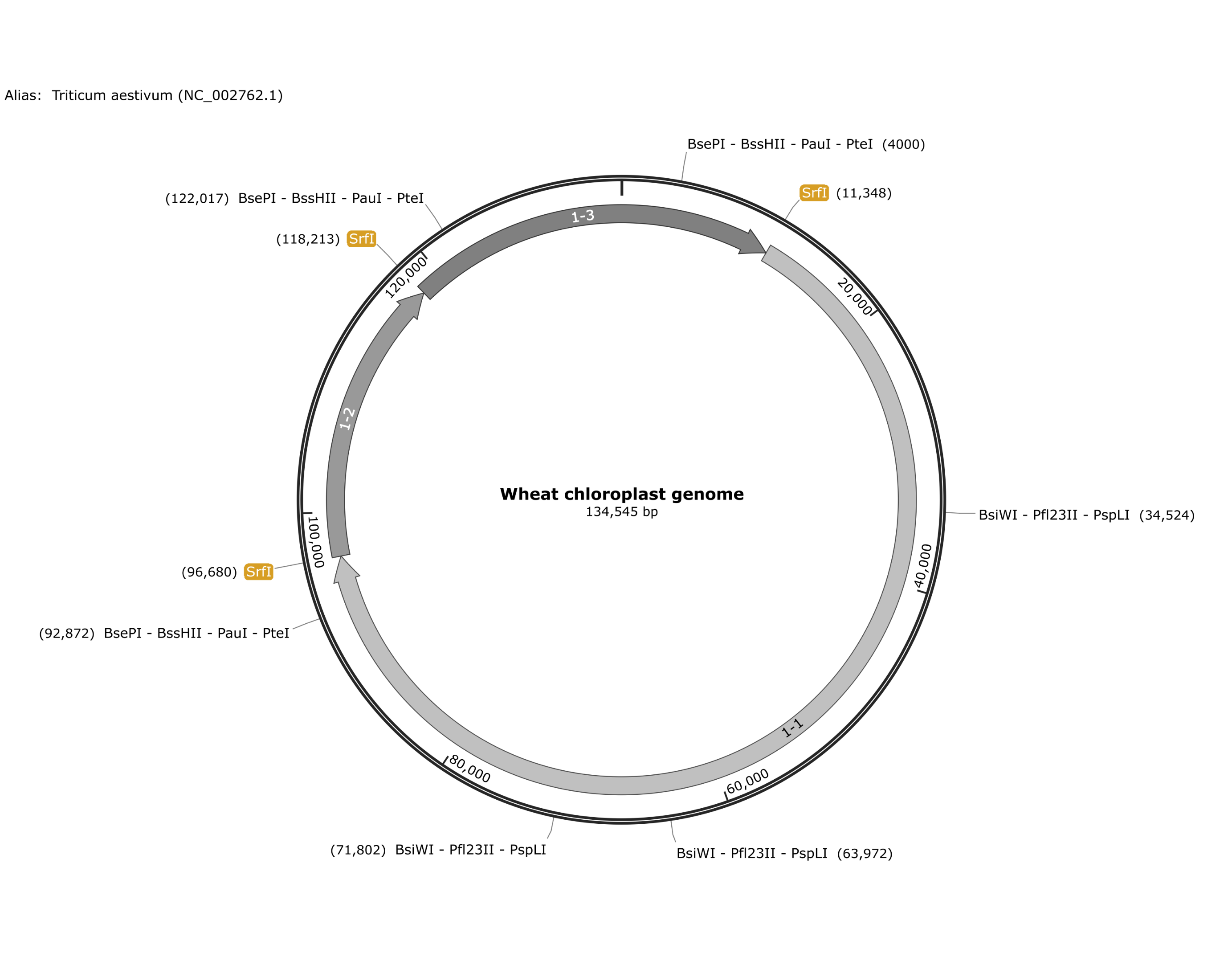

Supplement: Supplementary file 1 — Additional file 1: Figure S1. (a) Sample of crop leaves. Using Hydroponics with the repeated-short time scale of lighting, reduced the carbohydrate content of the leaves. Respectively, the labels (1 to 4) are Barley (3–4 leaf stage), Wheat (3–4 leaf stage), Maize (V2–V3 stage), and Soybean (V3–V4 stage). The yellow and white arrowheads represent younger (V2 stage) and older (V6 stage) age groups. The soybean older leaves (white line), before and after the V6 stage, the leaves have turned yellow and entered the leaf senescence development. Whereas, under natural conditions, the V6 stage of soybean is at the beginning of vegetative growth. (b) Chloroplast images. Soybean cell and chloroplast are distinguished by labels 1 and 2, respectively. The black arrowheads denote the cell, and the red arrowheads denote the chloroplast. Left bar = 20 μm, right bar = 50 μm. Figure S2. (a) Circular cpDNA extraction. The remaining gel (Lane 1, 2 and 3, red arrowhead) was stained with ethidium bromide before reconstructing the integrated gel to cut the elutable gel slices that contained the unstained circular cpDNA (Lane 4, 5 and 6). Subjecting to PFGE using the following procedure: Reverse Voltage Gradient: 4.5 V/cm, Int. Sw. Tm: 60 s, Fin. Sw. Tm:90 s, Included angle: 120°, Run Time: 18 h, Ramping factor: a=: ENTER. The HMW lambda ladder loaded into the far left. Attention circular DNA size cannot be marked up with a linearized DNA ladder because of the circular topology properties, whereas it can serve as a relative reference standard. (b) Recovering the circular cpDNA from gel slices using the D-tube Dialyzer. A supporting tray containing the unstained gel slice is built by placing the unstained gel slice into the D-Tube dialyzer. Filling the D-tube dialyzer with 5 mM Tris-HCl running buffer to the top of membranes. Avoid introducing air bubbles in the tube, as they interfere with electroelution. The optimum electroelution time must be determined according to the sample and gel c [file 13007_2023_1126_MOESM1_ESM.zip › Additional file 1 Figs - labels/Figure S3.tif]

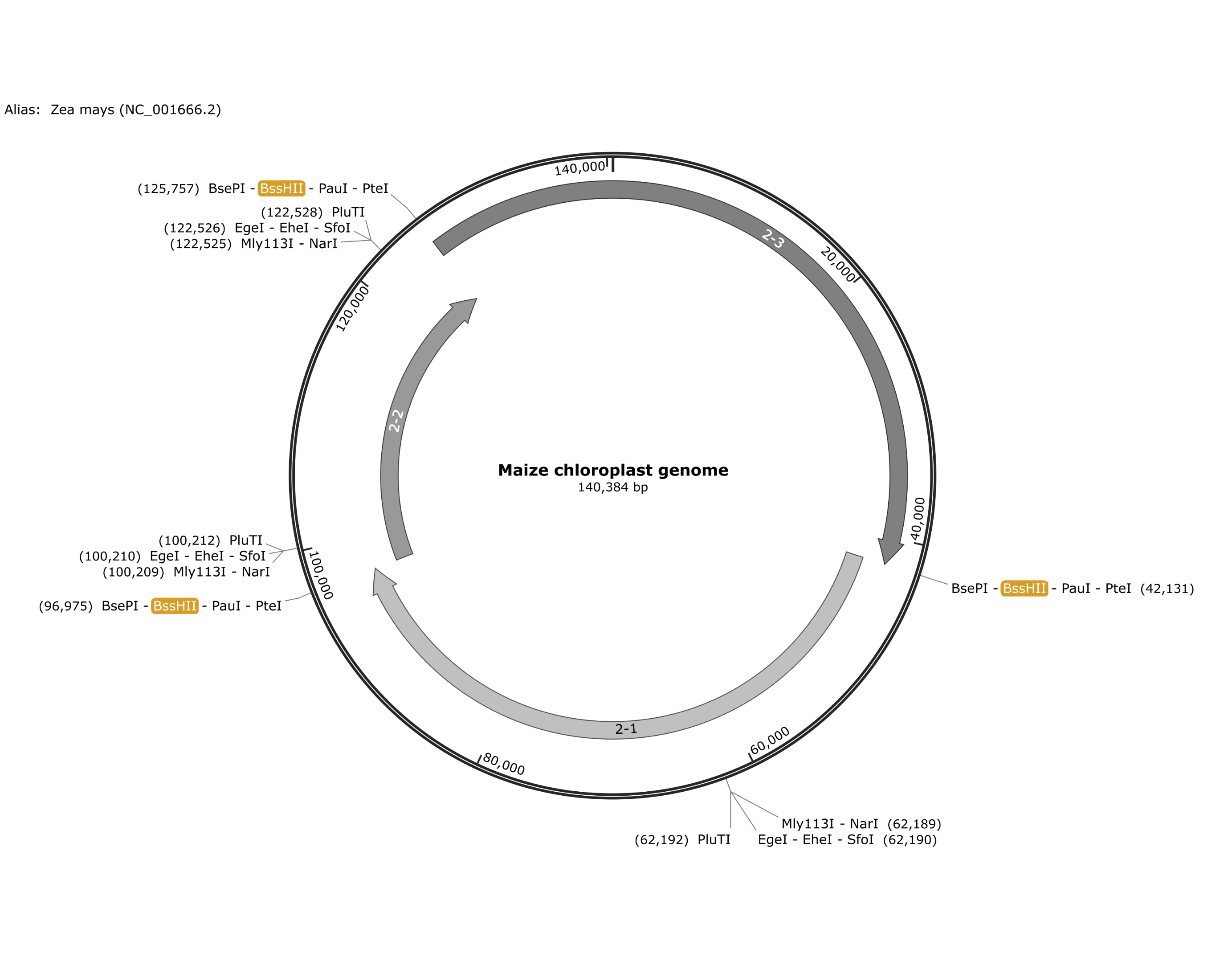

Supplement: Supplementary file 1 — Additional file 1: Figure S1. (a) Sample of crop leaves. Using Hydroponics with the repeated-short time scale of lighting, reduced the carbohydrate content of the leaves. Respectively, the labels (1 to 4) are Barley (3–4 leaf stage), Wheat (3–4 leaf stage), Maize (V2–V3 stage), and Soybean (V3–V4 stage). The yellow and white arrowheads represent younger (V2 stage) and older (V6 stage) age groups. The soybean older leaves (white line), before and after the V6 stage, the leaves have turned yellow and entered the leaf senescence development. Whereas, under natural conditions, the V6 stage of soybean is at the beginning of vegetative growth. (b) Chloroplast images. Soybean cell and chloroplast are distinguished by labels 1 and 2, respectively. The black arrowheads denote the cell, and the red arrowheads denote the chloroplast. Left bar = 20 μm, right bar = 50 μm. Figure S2. (a) Circular cpDNA extraction. The remaining gel (Lane 1, 2 and 3, red arrowhead) was stained with ethidium bromide before reconstructing the integrated gel to cut the elutable gel slices that contained the unstained circular cpDNA (Lane 4, 5 and 6). Subjecting to PFGE using the following procedure: Reverse Voltage Gradient: 4.5 V/cm, Int. Sw. Tm: 60 s, Fin. Sw. Tm:90 s, Included angle: 120°, Run Time: 18 h, Ramping factor: a=: ENTER. The HMW lambda ladder loaded into the far left. Attention circular DNA size cannot be marked up with a linearized DNA ladder because of the circular topology properties, whereas it can serve as a relative reference standard. (b) Recovering the circular cpDNA from gel slices using the D-tube Dialyzer. A supporting tray containing the unstained gel slice is built by placing the unstained gel slice into the D-Tube dialyzer. Filling the D-tube dialyzer with 5 mM Tris-HCl running buffer to the top of membranes. Avoid introducing air bubbles in the tube, as they interfere with electroelution. The optimum electroelution time must be determined according to the sample and gel c [file 13007_2023_1126_MOESM1_ESM.zip › Additional file 1 Figs - labels/Figure S4.tif]

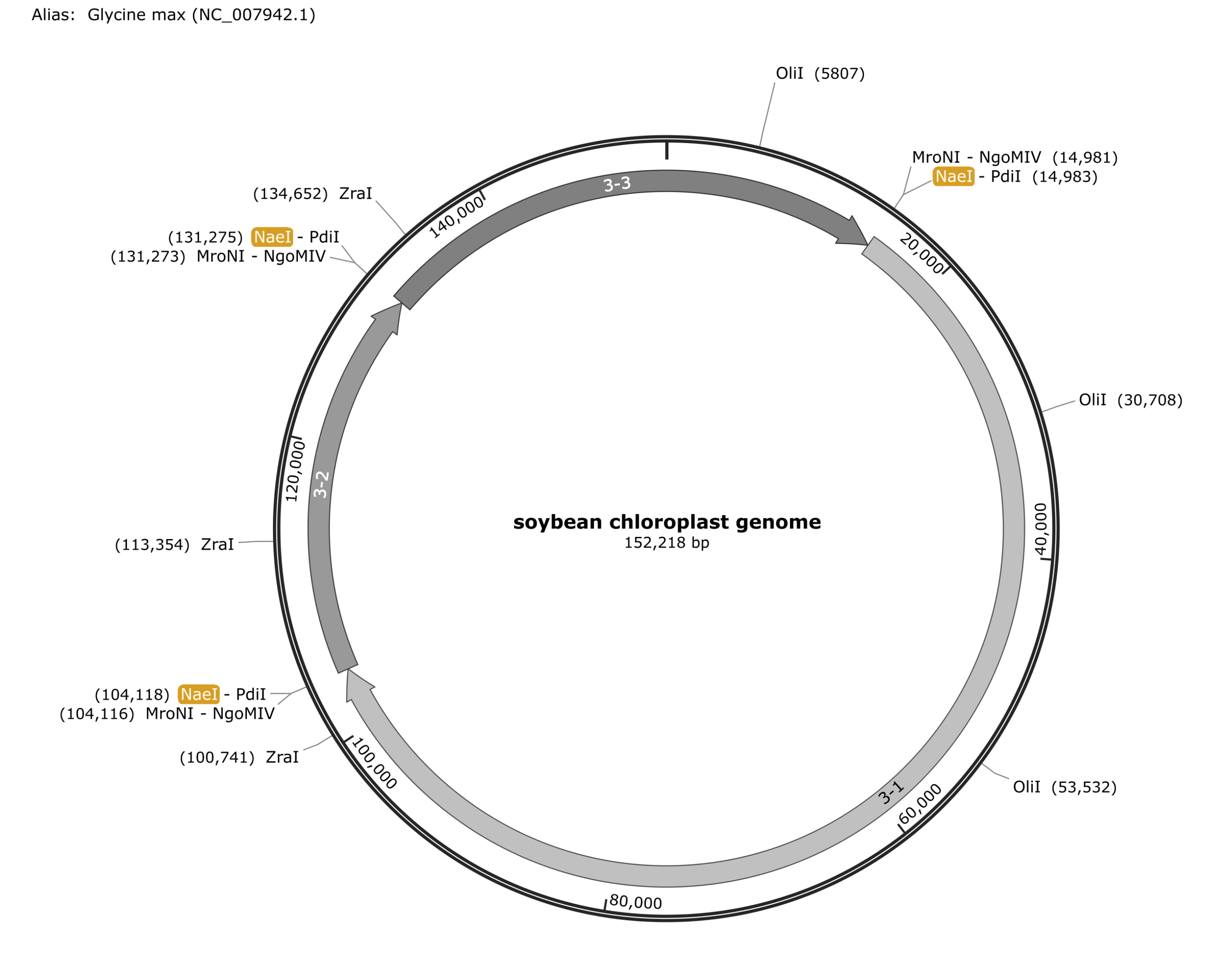

Supplement: Supplementary file 1 — Additional file 1: Figure S1. (a) Sample of crop leaves. Using Hydroponics with the repeated-short time scale of lighting, reduced the carbohydrate content of the leaves. Respectively, the labels (1 to 4) are Barley (3–4 leaf stage), Wheat (3–4 leaf stage), Maize (V2–V3 stage), and Soybean (V3–V4 stage). The yellow and white arrowheads represent younger (V2 stage) and older (V6 stage) age groups. The soybean older leaves (white line), before and after the V6 stage, the leaves have turned yellow and entered the leaf senescence development. Whereas, under natural conditions, the V6 stage of soybean is at the beginning of vegetative growth. (b) Chloroplast images. Soybean cell and chloroplast are distinguished by labels 1 and 2, respectively. The black arrowheads denote the cell, and the red arrowheads denote the chloroplast. Left bar = 20 μm, right bar = 50 μm. Figure S2. (a) Circular cpDNA extraction. The remaining gel (Lane 1, 2 and 3, red arrowhead) was stained with ethidium bromide before reconstructing the integrated gel to cut the elutable gel slices that contained the unstained circular cpDNA (Lane 4, 5 and 6). Subjecting to PFGE using the following procedure: Reverse Voltage Gradient: 4.5 V/cm, Int. Sw. Tm: 60 s, Fin. Sw. Tm:90 s, Included angle: 120°, Run Time: 18 h, Ramping factor: a=: ENTER. The HMW lambda ladder loaded into the far left. Attention circular DNA size cannot be marked up with a linearized DNA ladder because of the circular topology properties, whereas it can serve as a relative reference standard. (b) Recovering the circular cpDNA from gel slices using the D-tube Dialyzer. A supporting tray containing the unstained gel slice is built by placing the unstained gel slice into the D-Tube dialyzer. Filling the D-tube dialyzer with 5 mM Tris-HCl running buffer to the top of membranes. Avoid introducing air bubbles in the tube, as they interfere with electroelution. The optimum electroelution time must be determined according to the sample and gel c [file 13007_2023_1126_MOESM1_ESM.zip › Additional file 1 Figs - labels/Figure S5.tif]

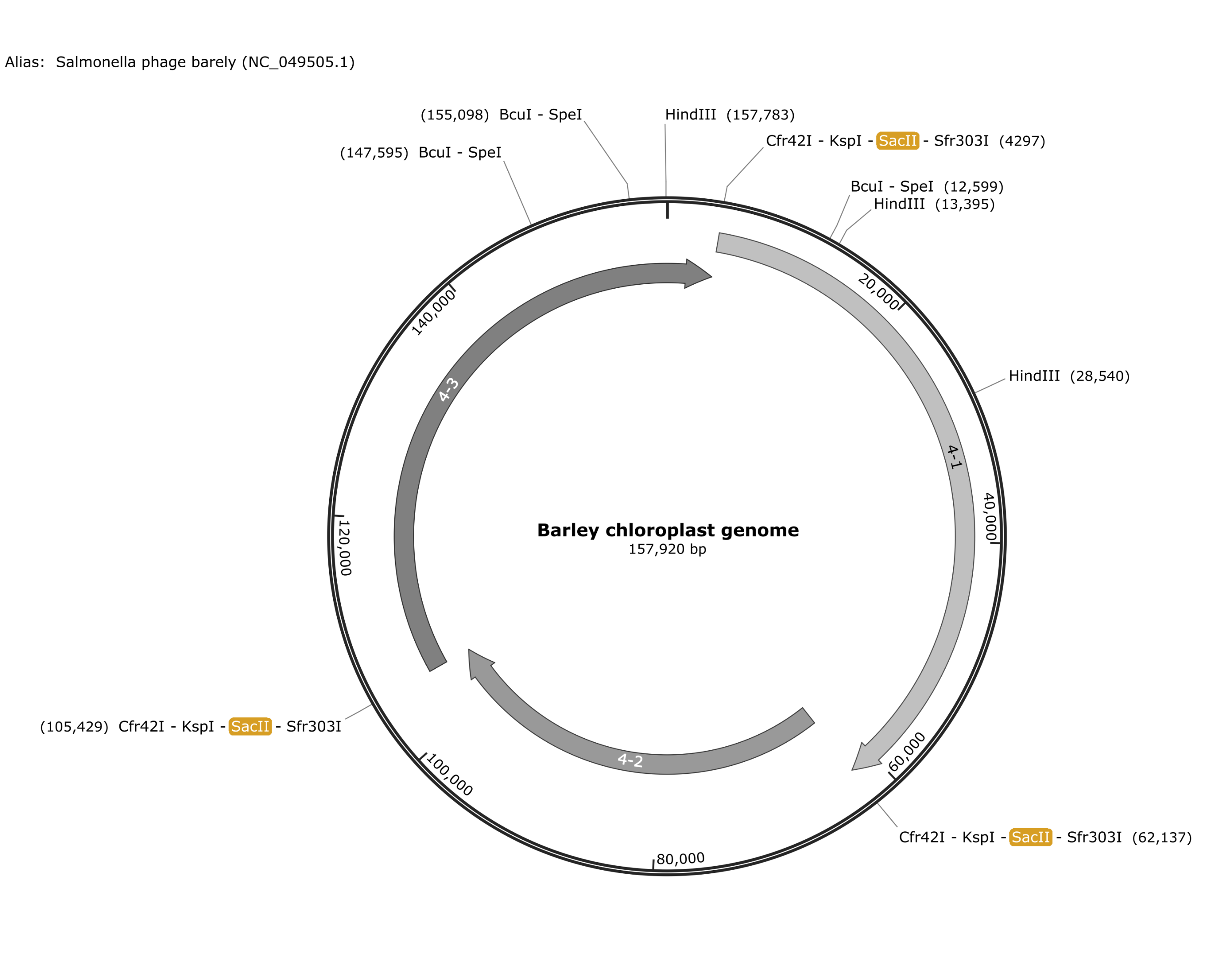

Supplement: Supplementary file 1 — Additional file 1: Figure S1. (a) Sample of crop leaves. Using Hydroponics with the repeated-short time scale of lighting, reduced the carbohydrate content of the leaves. Respectively, the labels (1 to 4) are Barley (3–4 leaf stage), Wheat (3–4 leaf stage), Maize (V2–V3 stage), and Soybean (V3–V4 stage). The yellow and white arrowheads represent younger (V2 stage) and older (V6 stage) age groups. The soybean older leaves (white line), before and after the V6 stage, the leaves have turned yellow and entered the leaf senescence development. Whereas, under natural conditions, the V6 stage of soybean is at the beginning of vegetative growth. (b) Chloroplast images. Soybean cell and chloroplast are distinguished by labels 1 and 2, respectively. The black arrowheads denote the cell, and the red arrowheads denote the chloroplast. Left bar = 20 μm, right bar = 50 μm. Figure S2. (a) Circular cpDNA extraction. The remaining gel (Lane 1, 2 and 3, red arrowhead) was stained with ethidium bromide before reconstructing the integrated gel to cut the elutable gel slices that contained the unstained circular cpDNA (Lane 4, 5 and 6). Subjecting to PFGE using the following procedure: Reverse Voltage Gradient: 4.5 V/cm, Int. Sw. Tm: 60 s, Fin. Sw. Tm:90 s, Included angle: 120°, Run Time: 18 h, Ramping factor: a=: ENTER. The HMW lambda ladder loaded into the far left. Attention circular DNA size cannot be marked up with a linearized DNA ladder because of the circular topology properties, whereas it can serve as a relative reference standard. (b) Recovering the circular cpDNA from gel slices using the D-tube Dialyzer. A supporting tray containing the unstained gel slice is built by placing the unstained gel slice into the D-Tube dialyzer. Filling the D-tube dialyzer with 5 mM Tris-HCl running buffer to the top of membranes. Avoid introducing air bubbles in the tube, as they interfere with electroelution. The optimum electroelution time must be determined according to the sample and gel c [file 13007_2023_1126_MOESM1_ESM.zip › Additional file 1 Figs - labels/Figure S6.tif]

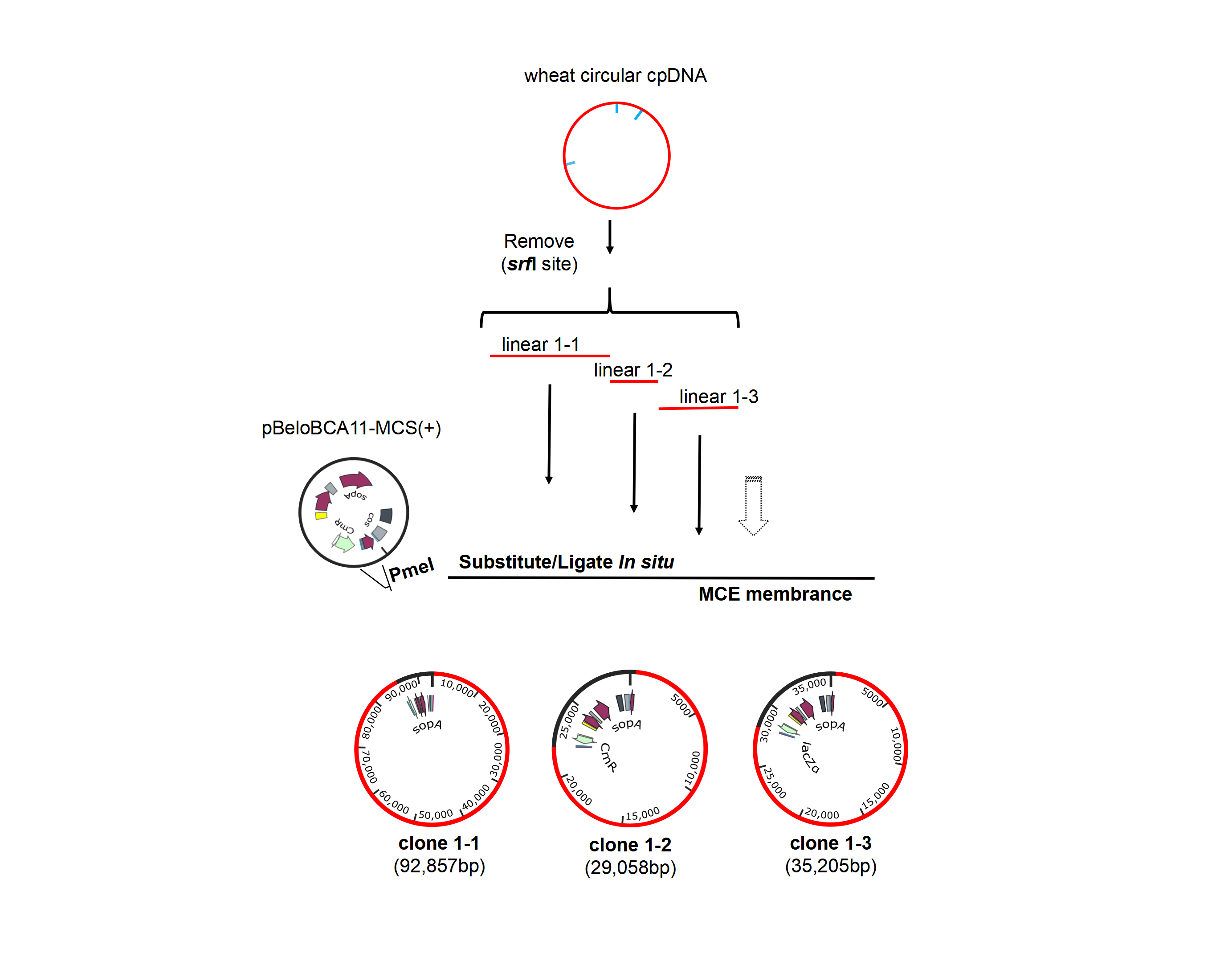

Supplement: Supplementary file 1 — Additional file 1: Figure S1. (a) Sample of crop leaves. Using Hydroponics with the repeated-short time scale of lighting, reduced the carbohydrate content of the leaves. Respectively, the labels (1 to 4) are Barley (3–4 leaf stage), Wheat (3–4 leaf stage), Maize (V2–V3 stage), and Soybean (V3–V4 stage). The yellow and white arrowheads represent younger (V2 stage) and older (V6 stage) age groups. The soybean older leaves (white line), before and after the V6 stage, the leaves have turned yellow and entered the leaf senescence development. Whereas, under natural conditions, the V6 stage of soybean is at the beginning of vegetative growth. (b) Chloroplast images. Soybean cell and chloroplast are distinguished by labels 1 and 2, respectively. The black arrowheads denote the cell, and the red arrowheads denote the chloroplast. Left bar = 20 μm, right bar = 50 μm. Figure S2. (a) Circular cpDNA extraction. The remaining gel (Lane 1, 2 and 3, red arrowhead) was stained with ethidium bromide before reconstructing the integrated gel to cut the elutable gel slices that contained the unstained circular cpDNA (Lane 4, 5 and 6). Subjecting to PFGE using the following procedure: Reverse Voltage Gradient: 4.5 V/cm, Int. Sw. Tm: 60 s, Fin. Sw. Tm:90 s, Included angle: 120°, Run Time: 18 h, Ramping factor: a=: ENTER. The HMW lambda ladder loaded into the far left. Attention circular DNA size cannot be marked up with a linearized DNA ladder because of the circular topology properties, whereas it can serve as a relative reference standard. (b) Recovering the circular cpDNA from gel slices using the D-tube Dialyzer. A supporting tray containing the unstained gel slice is built by placing the unstained gel slice into the D-Tube dialyzer. Filling the D-tube dialyzer with 5 mM Tris-HCl running buffer to the top of membranes. Avoid introducing air bubbles in the tube, as they interfere with electroelution. The optimum electroelution time must be determined according to the sample and gel c [file 13007_2023_1126_MOESM1_ESM.zip › Additional file 1 Figs - labels/Figure S7.tif]

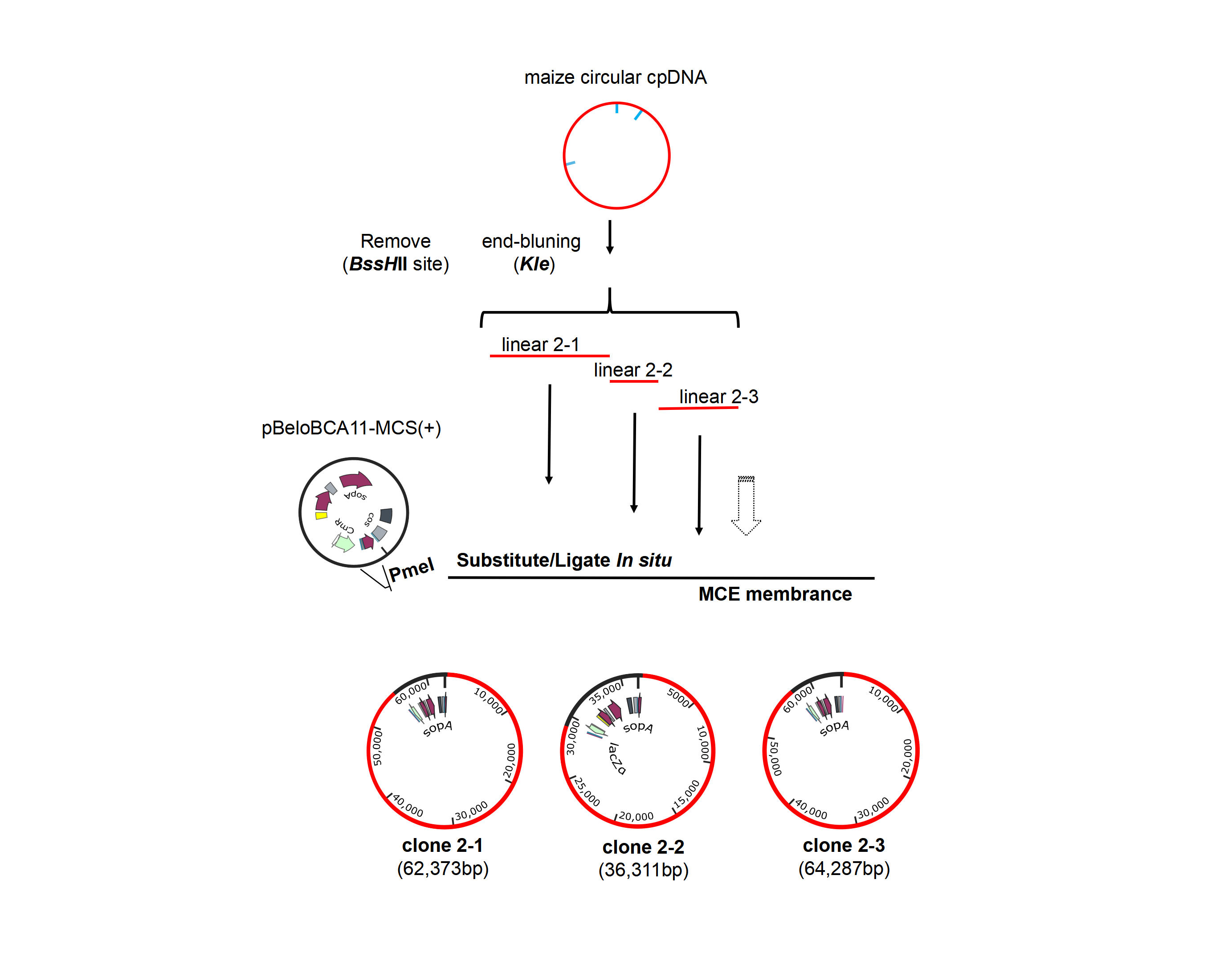

Supplement: Supplementary file 1 — Additional file 1: Figure S1. (a) Sample of crop leaves. Using Hydroponics with the repeated-short time scale of lighting, reduced the carbohydrate content of the leaves. Respectively, the labels (1 to 4) are Barley (3–4 leaf stage), Wheat (3–4 leaf stage), Maize (V2–V3 stage), and Soybean (V3–V4 stage). The yellow and white arrowheads represent younger (V2 stage) and older (V6 stage) age groups. The soybean older leaves (white line), before and after the V6 stage, the leaves have turned yellow and entered the leaf senescence development. Whereas, under natural conditions, the V6 stage of soybean is at the beginning of vegetative growth. (b) Chloroplast images. Soybean cell and chloroplast are distinguished by labels 1 and 2, respectively. The black arrowheads denote the cell, and the red arrowheads denote the chloroplast. Left bar = 20 μm, right bar = 50 μm. Figure S2. (a) Circular cpDNA extraction. The remaining gel (Lane 1, 2 and 3, red arrowhead) was stained with ethidium bromide before reconstructing the integrated gel to cut the elutable gel slices that contained the unstained circular cpDNA (Lane 4, 5 and 6). Subjecting to PFGE using the following procedure: Reverse Voltage Gradient: 4.5 V/cm, Int. Sw. Tm: 60 s, Fin. Sw. Tm:90 s, Included angle: 120°, Run Time: 18 h, Ramping factor: a=: ENTER. The HMW lambda ladder loaded into the far left. Attention circular DNA size cannot be marked up with a linearized DNA ladder because of the circular topology properties, whereas it can serve as a relative reference standard. (b) Recovering the circular cpDNA from gel slices using the D-tube Dialyzer. A supporting tray containing the unstained gel slice is built by placing the unstained gel slice into the D-Tube dialyzer. Filling the D-tube dialyzer with 5 mM Tris-HCl running buffer to the top of membranes. Avoid introducing air bubbles in the tube, as they interfere with electroelution. The optimum electroelution time must be determined according to the sample and gel c [file 13007_2023_1126_MOESM1_ESM.zip › Additional file 1 Figs - labels/Figure S8.tif]

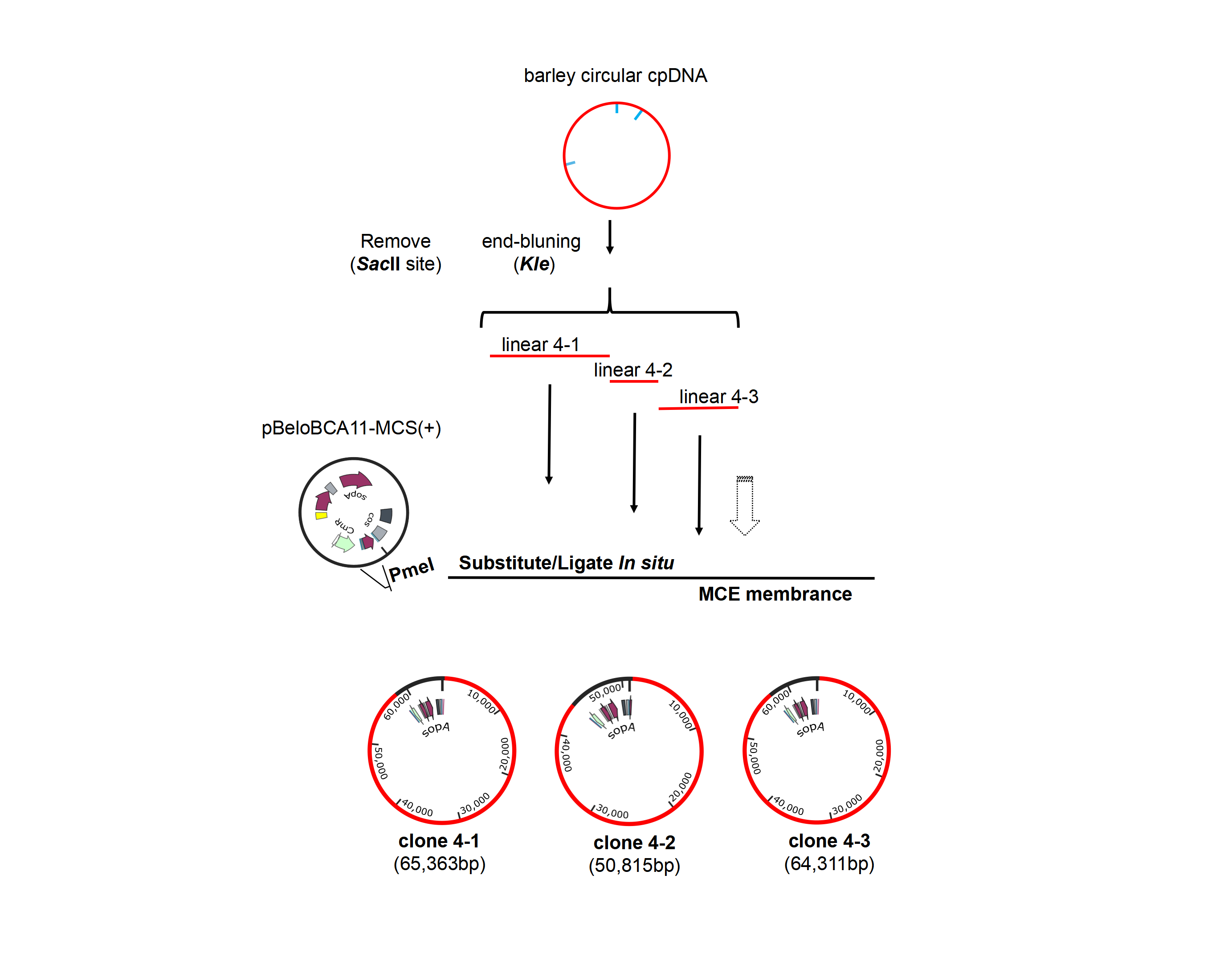

Supplement: Supplementary file 1 — Additional file 1: Figure S1. (a) Sample of crop leaves. Using Hydroponics with the repeated-short time scale of lighting, reduced the carbohydrate content of the leaves. Respectively, the labels (1 to 4) are Barley (3–4 leaf stage), Wheat (3–4 leaf stage), Maize (V2–V3 stage), and Soybean (V3–V4 stage). The yellow and white arrowheads represent younger (V2 stage) and older (V6 stage) age groups. The soybean older leaves (white line), before and after the V6 stage, the leaves have turned yellow and entered the leaf senescence development. Whereas, under natural conditions, the V6 stage of soybean is at the beginning of vegetative growth. (b) Chloroplast images. Soybean cell and chloroplast are distinguished by labels 1 and 2, respectively. The black arrowheads denote the cell, and the red arrowheads denote the chloroplast. Left bar = 20 μm, right bar = 50 μm. Figure S2. (a) Circular cpDNA extraction. The remaining gel (Lane 1, 2 and 3, red arrowhead) was stained with ethidium bromide before reconstructing the integrated gel to cut the elutable gel slices that contained the unstained circular cpDNA (Lane 4, 5 and 6). Subjecting to PFGE using the following procedure: Reverse Voltage Gradient: 4.5 V/cm, Int. Sw. Tm: 60 s, Fin. Sw. Tm:90 s, Included angle: 120°, Run Time: 18 h, Ramping factor: a=: ENTER. The HMW lambda ladder loaded into the far left. Attention circular DNA size cannot be marked up with a linearized DNA ladder because of the circular topology properties, whereas it can serve as a relative reference standard. (b) Recovering the circular cpDNA from gel slices using the D-tube Dialyzer. A supporting tray containing the unstained gel slice is built by placing the unstained gel slice into the D-Tube dialyzer. Filling the D-tube dialyzer with 5 mM Tris-HCl running buffer to the top of membranes. Avoid introducing air bubbles in the tube, as they interfere with electroelution. The optimum electroelution time must be determined according to the sample and gel c [file 13007_2023_1126_MOESM1_ESM.zip › Additional file 1 Figs - labels/Figure S9.tif]
