# Supplementary material for: A novel in-situ-process technique constructs whole circular cpDNA library
Source: Plant Methods. 2024 Jan 3;20:2. doi: 10.1186/s13007-023-01126-7 (PMC10763311; doi:10.1186/s13007-023-01126-7)
Supplement: Supplementary file 2 — Additional file 2: Table S1. A summary of the basic components and procedures for optimizing chloroplast and cpDNA extraction. [file 13007_2023_1126_MOESM2_ESM.docx]

**Table S1** A summary of the basic components and procedures for optimizing chloroplast and cpDNA extraction

| Chloroplast isolation | | | | | | | | | | | | cpDNA extraction | | | | | Reference |
| --- | --- | --- | --- | --- | --- | --- | --- | --- | --- | --- | --- | --- | --- | --- | --- | --- | --- |
| Homogenization | | | | | | | | Centrifugation | | | | Iysis | | | | Extraction |  |
| Sampling | Medium | | | Rupture | | | | Crude chloroplast | | Purify the chloroplasts | |  |  |  |  |  |  |
| *spinach, beet* | sea sand, 0.4M sucrose, 0.02 M tris, 0.01 M NaCI, 0.005 M EDTA (pH 8.0) | | | Cheesecloth | | | | 1000 g/20 min | | | | 0.15 M NaCI, 0.10 M EDTA, 1% SDS ( pH 7.4), Ribonuclease | | | | CsCl density gradient. | Chun et al. [16] |
| *C. reinhardi* | W medium | | | 3000 psi | | | | sucrose density gradient | | sucrose density gradient | | 0.15 M NaCI, 0.10 M EDTA, 2% IPC-SDS | | | | DNA-CsCL mixtures | Sager and Ishida. [17] |
| *tobacco* | Honda medium | | | Chop, mesh cloth | | | | 1000 g/15 min | | sucrose density gradient | | SSC: 0.15 M NaCI, 0.015 M sodium cutrate, Ribonuclease | | | | CsCl density gradient | Tewari and Wildman [18] |
| *C. reinhardtii* | 0.15 M NaCI, 0.015 M sodium citrate  (pH 7.0), 50% sucrose | | | - | | | | | | | | 2.5% sodium dodecyl sarcosinate, 25 μg/mL pancreatic RNases, 20 u/mL T_1_ ribonucleases, 100 μg/mL pronase | | | | lyophilization, CsCl density gradient | Chiang and Sueoka [19] |
| *C. reinhardtii* | 0.15 M NaCI, 0.1 M EDTA (pH 8.0), 1.35% sucrose | | | | 2% sodium dodecyl sarcosinate, 2% sodium lauryl sulfate, 5% Triton X-100, 1% deoxycholate, T_1_ ribonucleases, pancreatic RNases | | | | | | | | | | | CsCl density gradient | Chiang [20] |
| *C. reinhardtii* | 0.15 M NaCI, 0.015 M sodium citrate  (pH 7.0), 50% sucrose | | | - | | | | 2% sodium dodecyl sarcosinate, 2% sodium lauryl sulfate, 5% Triton X-100, 1% deoxycholate, Pronase, RNase | | | | | | Chloroform/Isoamylalcohol (24:1), CsCl density gradient | | | Bastia et al. [21] |
| *Antirrhinum majus, et al..* | 0.3% PVP plus mannitol medium (w/v) | | grinding,  20 μm nylon | 2000 g/30s | | | 10 mM MgSO_4_ plus before centrifugation (2000g/30-45 s) | | | | 100 μg/mL DNAase I, 30 μg/mL phosphodiesterase, saline/EDTA, Tris/EDTA, 2.5% sodium sarcosinate (DNAase-free Pronase) | | | | CsC1 gradient, CsC1/ethidium bromide gradient | | Herrmann et al. [22] |
| *Spinach* | G-R medium | grinding, miracloth | | | | 6000 rpm/x s | | | Standard gradients: 1.05 g PEG6000, 0.35 g BSA, 0.35 g Ficoll, 35 mL silica sol | | | | | | | 7000 rpm/15 min | Morgenthaler et al. [23] |
| *Pea* | 0.33 M D (-) sorbitol, 0.1% (W/V) BSA, 50 mM HEPES (pH 6.5) | | | grinding, miracloth | | | | density gradient (5000g/15 s) | | sucrose density-gradient or 'washing' step | | | - | | | | Elias and Givan [24] |

| Continued to table S1 | | | | | | | | | | | | | | | | | | | | | | | | | | | | | | | | | | | | | | | | | | | | | | | | | | | | | | | | | | | | |
| --- | --- | --- | --- | --- | --- | --- | --- | --- | --- | --- | --- | --- | --- | --- | --- | --- | --- | --- | --- | --- | --- | --- | --- | --- | --- | --- | --- | --- | --- | --- | --- | --- | --- | --- | --- | --- | --- | --- | --- | --- | --- | --- | --- | --- | --- | --- | --- | --- | --- | --- | --- | --- | --- | --- | --- | --- | --- | --- | --- | --- |
| *S. oligorrhiza* | 0.45 M mannitol, 7 mM EDTA, 5 mM β-ME, 50 mM Tris-HCI (pH 8.0) | | | | grinding, 30/200 μm nylon gauze | | | | | | | | | | | | 2500 g/1.45 min | | | | | | | | | | | | | | | | | saline/EDTA, ET buffer, 2% (W/V) sodium sarkosinate | | | | | | | | | | | | | | | | | | | CsCl density gradient (1500 g/20 min) | | | | | | | van Ee et al.[25] |
| *spinach* | 0.33 M mannitol, 30 mM Mops-NaCI  (pH 7.8), 2 mM EDTA, 0.15% BSA | | | | grinding, muslin | | | | | | | 2200 g/30 s | | | | | | | | | | | | | Percoll gradient | | | | | | | | | - | | | | | | | | | | | | | | | | | | | | | | | | | | Mourioux and Douce [26] |
| *pea* | 50 mM Hepes (pH 7.5), 0.33 M sorbitol, 0.1% BSA | | | | Cheesecloth | | | | | | | 2500 g/2 min | | | | | | | | | | | | | Percoll gradients | | | | | | | | | - | | | | | | | | | | | | | | | | | | | | | | | | | | Cline et al. [27] |
| *pea* | 50 mM Tris (pH 8.0), 25 mM EDTA, 10 mM ME, 0.1% BSA, 1.25 M NaCI | | | | grinding, 20 μm nylon gauze | | | | | | | 1500 g/5 min | | | | | | | | | | | | | 1500 g/5 min | | | | | | | | | 0.5% sodium dodecyl sulfate, 2% sarkosyl, 50 μg/mL proteinase K | | | | | | | | | | | | | | | | | | | | Phenol/Chlroform/Ethanol (25:24:1) | | | | | | Bookjans et al. [28] |
| *rice* | 50 mM Tris-HCl (pH 8.0), 0.35 M sucrose, 7 mM EDTA, 5 mM 2-ME, 0.1% BSA | | | | | blended,cheese cloth, miracloth | | | | | | | | 1000 g/10 min | | | | | | | | | | | 20%/45%/60% sucrose gradients (2000 g/30 min) | | | | | | | | | | | | | | 50 mM Tris-HC1(pH 8.0), 20 mM EDTA, 3% sodium dodecylsarcosinate, Pronase E (10 mg/mL) | | | | | | | | | | | | | | | Phenol/phenol-chloroform (1 : 1) | | | | | | Hirai et al. [29] |
| *tabacum* | 30 mM MOPS (pH 7.2), 0.3 M sorbitol, 3 mM EDTA, 8 mM 2-mercapto ethanol, 0.2% BSA | | | | | | | grinding,  20 μm nylon mesh | | | | | | | | | | | 1300 g/10 min | | | | | | | | | | Self-generated Percoll gradients | | | | | | | | | 150 mM NaCI, 15 mM EDTA, 40 mM Tris-HCI  (pH 7.9), 2% sarkosyl, 50 μg/mL proteinase K | | | | | | | | | | | | | | | Chloroform / Isoamylalcohol (24:1) | | | | | | | Pay and Smith [30] |
| *Spinach* | 1 mM sodium pyrophosphate, 50 mM HEPES, 0.33 M sorbitol, 10 mM dithiotheritol, 1 mM MnCI, 2mM EDTA (pH 6.8) | | | | | | | | | | 6000 g/30 s | | | | | | | | | 1% LMP: 0.33 M sorbitol,25 mM citrate hydrochloride (pH 7.0), 90 mM 2-ME, 125 mM EDTA.  lysis buffer: 450 mM EDTA, 1% sodium *N*-laurylsarcosinate, 10 mM Tris-HCI (pH 8.0),15 mg proteinase K | | | | | | | | | | | | | | | | | | | | | | | | | | | | | | | | | | | | | | PFGE | | Deng et al. [3] |
| *Rice* | 0.5% cellulase Onozuka RS, 1% pectinase, C salt solution, 0.8 M mannitol (pH 5.8) | | | grinding,  45 μm nylon mesh | | | | | | | 800 g/5 min | | | | | | | | | 0.5% LMP: C salt solution, mannitol, PBS buffer. Lysis buffer: 1 mg/mL proteinase K in NDS buffer (100 mM EDTA, 100 mM Tris-HCI, 1% sodium-laurylsarcosine (pH 9.5) | | | | | | | | | | | | | | | | | | | | | | | | | | | | | | | | | | | | | | PFGE | | Cuzzoni et al. [31] |
| *watermelon, pea, cauliflower* | G-R medium | | grinding,miracloth | | | | | | | | | 6000 rpm /x s | | | | | | | | | | | | | Percoll gradients | | | | | | | | | 100-200 μg/mL DNAse  Agarose insert:200 μg/mL proteinase K | | | | | | | | | | | | | | | PFGE:5V/cm, 120s pulse time, 1.5% agarose | | | | | | | | | | | Bendich and Smith [4] |
| *watermelon, pea* | G-R medium | | grinding, miracloth | | | | | | | | | 6000 rpm/x s | | | | | | | | | | | | | Percoll gradients | | | | | | | | | | | DNase. Agarose inserts: proteinase K, sarkosyl | | | | | | | | | | | | | | | | | | PFGE | | | | | | Bendich [14] |
| *Arabidopsis* | MS medium, enzyme solution:1% cellase Onozuka R, 0.1% Macerozyme, 0.4% M sucrose | | | | swirl, 100 μm nylon mesh | | | | | | | 16% sucrose in  1/10 x CPW-16 (200 rpm/5 min) | | | | | | | | | | | | | | | | | | | salt solution W5 (600 rpm/5 min) | | | | | | | | 1% FMC: CPW-16, ATA. Lysis buffer: 0.5 mg/mL, proteinase K, 100 mM EDTA (pH8.0) , 1% sarkosyl, 2 mM ATA | | | | | | | | | | | | | | | | | | PFGE | | | Bancroft et al. [32] |
| *Arabidopsis* | 9 mL digestion medium, 70 g/L sorbitol, 20 g/L glucose, 15 g/L glycine, 0.07% MES (pH 8.0), 0.0008% bromocesol blue, 0.001% Tween-80, 1 mg/L naphtaleneacetic acid, 1mg/L benzylaminopurine, 100 mg/L myo-inositol, 0.01 mg/L niacin, 1 mg/L calcium panthotenate, 1mg/L pyridoxin, 1 mg/L thiamine-HCI | | | | | | | | | | | | | | | | | | | | | | | | enzymes: 1 mL of 0.2% macerozyme R-10, 1% cellulase Onozuke, 50 uL 10% Driselase, 80/140 μm steel sieves | | | | | | | | | | | | | | | | | 300 g/10 min | | | 1% LMP: 0.3 M CaCI_2_.  Lysis buffer: NDS buffer(10 mM Tris-HCI, 0.5M EDTA, 1% lauroylsarcosine pH 8.0), 2 mg/mL proteinase K | | | | | | | | | | | | PFGE | | | Creusot et al. [33] |
| Continued to table S1 | | | | | | | | | | | | | | | | | | | | | | | | | | | | | | | | | | | | | | | | | | | | | | | | | | | | | | | | | | | | |
| *N.Tabacum* | 0.33 M sorbitol, 50 mM Hepes-KOH  (pH 8.0), 2 mM EDTA | | | | grinding, miracloth | | | | | | 4000g/1 min | | | | | | | | | | | 35%/80% Percoll gradients (2500 g/5 min) | | | | | | | | | | | | 0.5% LMP: PBSA solution.  Lysis buffer: 0.5 M EDTA (pH 8.0), 1% SDS, 0.5 mg/mL Proteinase K | | | | | | | | | | | | | | | | | | | | | | | PFGE | | | Backert et al. [5] |
| *Sunflower* | STE buffer: 400 mM sucrose, 50 mM Tris (pH 7.8), 20 mM EDTA-Na2, 0.2% BSA, 0.2% β-ME | | | | | | | | grinding, dense nylon | | | | | | | | | 200 g, 3700 g | | | | | | ST buffer: 400 mM sucrose, 50 mM Tris pH 7.8, 0.1% BSA. 25 μg/mL DNase-1, 0.02 M Mg-acetate (Mg-chloride, Mg-sulphate) | | | | | | | | | | | | | | | | | | | | | | | | TEN buffer, 1% SDS | | | | | | Phenol/Chlorofom,  Ethanol | | | | | | Triboush et al. [34] |
| *Arabidopsis* | 0.35 M sorbitol, 50 mM Tris-HC1, (pH 8.0), 5 mM disodium EDTA, 0.1% BSA , 15 mM β-ME | | | | | | grinding, cheesecloth, miracloth | | | | | | | | | 1000 g/10 min | | | | | | | | | | sucrose step-gradient | | | | | | | | | | | 5% (w/v) sodium sarcosinate, 50 mM Tris-HC1 (pH 8.0),  25 mM disodium EDTA | | | | | | | | | | | | | | EtBr plus CsCl density-gradient  (189000 g/12-24 h) | | | | | | | | | Mourad [35] |
| *red alga* | 20 mM Tris-HCl (pH 7.6), 5 mM MgCl_2_, 5 mM KCl, 5 mM EGTA,180 mM sucrose | | | | | | | | | | | | | | | | | | | | | | | | | | 1500 psi | | | | | | | | | | | | | | | | 100 mg/mL DNase I, Percoll gradients | | | | | | | | | - | | | | | | | | Miyagishima et al. [36] |
| *Cenchrus ciliaris L, Poa alpina L, et.al.* | 0.35 M sorbitol, 0.1 M Tris, 5 mM EDTA (pH 7.8), 0.5% sodium bisulfite.  ( 0.5% sodium bisulfite) | grinding,  33 μm mesh | | | | | 4000 g/25 min | | | | ① 4 mL NET buffer (100 mM NaCl, 80 mM Tris-HCl, 30 mM EDTA (pH 8.0))  ② 1 mL 20% Triton X-100, 200 uL 2-β-ME, 150 uL pronase(10 mg/mL), 7 uL proteinase K (20 mg/mL), 160 uL 20% SDS  ③ 2.2 mL CTBA buffer, 100 uL 2-β-ME, 0.05 g PVP  ④chloroform-isoamyl alcohol (24:1) | | | | | | | | | | | | | | | | | | | | | | | | | | | | | | | | | | | | | | | | | | | ①4800 g/10 min  ② 4800 g/10 min  ③ 4000 g/5 min  ④ 9000 g/10 min | | | | | | Mariac et al. [37] |
| *Tobacco, Arabidopsis, pea* | 0.45 M sorbitol, 50 mM Tris (pH 7.6), 5 mM EDTA, 0.2%(W/V) BSA, 1.0% polyvinypyrrolidone-362, 0.025% spermine, 0.025% spermidine, β-ME | | | | | | | | | | | | | | Grinding, cheesecloth, miracloth | | | | | | | | | | | | | | | 4000g /x s | | | | | 10 mM MgCI_2_, 25 μg/mL DNase, EDTA, Percoll gradients | | | | | | | | | | | 1% LMP: wash buffer. Lysis buffer: 2% sarkosyl, 0.45 M EDTA, 10 μg/mL proteinase K | | | | | | | | | | PFGE | | | | Lilly et al. [6] |
| *Maize, M. truncatula* | 330 mM sorbitol, 2 mM EDAT, 1 mM MgCI2, 0.1% BSA. 50 mM N-Tris (hydroxymethyl) methylglycine/KOH (pH 7.9) | | | | | | | | | | | | grinding,cheesecloth, miracloth. | | | | | | | | | | | | | | | DNase | | | | | Percoll gradient | | | | | | | | Lysis buffer: 0.47M EDTA (pH 8.0), 1% sarkosyl, 200μg/mL proteinase K | | | | | | | | | | | | | | PFGE | | | | | Bendich [38] |
| *Lactuca* | isolation buffer: 1.25M NaCI, 5mM EDTA, 1% BSA, 10mM 2-ME, 5% PVP-40, 50mM Tris-HCI (pH 8.0) | | | | | | | | | grinding, cheesecloth, miracloth | | | | | | | | | | | | | 1000 g/15 min | | | | | | | | | Sucrose step-gradient | | | | | | | | Pronase, 5 x lysis buffe (20% sarcosyl, 50 mM Tris(pH 8.0), 25 mM EDTA) | | | | | | | | | | EtBr plus CsCl density gradient (65000 rpm/6-8 h) | | | | | | | | | | Jansen et al. [39] |
| *Tobacco, Maize, Wheat* | SGB buffer: 0.33 M sorbitol, 50 mM HEPES (pH 7.6), 1 mM MgCI_2_, 2 mM EDTA, 0.1% BSA, 1% polyvinylpyrrolidine-40. HS buffer: 1.25 M NaCI, 40 mM HEPES (pH 7.6), 2 mM EDTA, 0.1% BSA, 0.1% β-ME | | | | | | | | | | | | | | | | | | | | grinding, miracloth | | | | | | SDB buffer: 0.33 M sorbitol, 20 mM HEPES (PH 7.6), 1 mM MgCI_2_, 2 mM EDTA, 0.1% BSA, (200 μg/mL DNase in 10 mM MgCI_2_) | | | | | | | | | | | | | | | | | Percoll gradient | | | lysis buffer: 40 mM EDTA  (pH 8.0), 1% sarkosyl, 200 μg/mL proteinase K | | | | | | | | | | | | PFGE | Shaver et al. [10] |

| Continued to table S1 | | | | | | | | | | | | | | | | | | | | | | | | | | | | | | |
| --- | --- | --- | --- | --- | --- | --- | --- | --- | --- | --- | --- | --- | --- | --- | --- | --- | --- | --- | --- | --- | --- | --- | --- | --- | --- | --- | --- | --- | --- | --- |
| *Green alga* | HEPES-KOH buffer: *N*-(2-hydroxyethyl) piperazine *N'* (2-ethane-sulfonic acid), isoascorbic acid (pH 7.0) | | | | | | | | | | | | | | | | 80 psi | | | | 750 g/x min | | | Percoll gradient (4200 g/15 min) | | | | | - | Moroney et al. [40] |
| *Lolium perenne* | 1.25 M NaCI, 50 mM Tris-HCI (pH 8.0), 7 mM EDTA, 5% PVP-40, 1% BSA, 1 mM DTT (1 mM 2-ME) | | | | grinding, miracloth | | | | 1366 g/20 min | | | | | 30%/52% sucrose gradient (2500 g/5 min, 2600 g/14.5 h, 36500 g/1 h) | | | | | | | | Lysis buffer: 10 mM Tris-CHI (pH 8.0), 5 mM EDTA, 10 mg/mL Proteinase K (2-ME) | | | | | Phenol/Chloroform/Isoamyl-alcohol (25:24:1) | | | Diekmann et al. [41] |
| *Arabidopsis* | 1.25 M NaCI, 2 mM EDTA, 0.1%(W/V) BSA, 0.1%(V/V) β-ME (antifoam), 40 mM (HEPES)-KOH (pH 7.6) | | | grinding, mirachoth | | 12000-16000 g/20 s (3000 g/5 min) | | | | | | 30%/70% Percoll gradient (12000-16000 g/10 min), (1500 g/30 min) | | | | | | | Lysis buffer III: SDB buffer (0.33 M sorbitol, 20 mM HEPES-KOH (pH 7.6), 1 mM MgCI_2_, 2 mM EDTA, 0.1%(W/V) BSA), 1% SDS, 2.5 mM EDTA, 200 μg/mL proteinase K | | | | | | | | | | 12000 g/10 min | Rowan and Bendich [42] |
| *green alga* | 400 mM sucrose, 50 mM Tris(pH 7.8), 20 mM EDTA, 0.2% BSA, 0.2% β-ME | | | grinding, cheese, cloth | | | | 800 g/4 min | | | | | 10% / 60% sucrose gradient (150000 g/90 min) | | | | | 50 mM Tris (pH 8.0), 25 mM EDTA, 2% SDS, 50 mg/mL proteinase K (10000 g/15 min) | | | | | | | | CsCl density  Gradient (240000 g/38 h) | | | | Lü et al. [43] |
| 1. *Brachyantha, L.japonica, P. utihis* | A method: 1.25 M NaCl, 0.25 M ascorbic acid, 10 mM sodium metabisulfite, 0.0125 M Borax, 50 mM Tris-HCl (pH 8.0), 7 mM EDTA, 1% PVP-40 (w/v), 0.1% BSA (w/v), 1 mM DTT  B method: 1.25 M NaCl, 50 mM Tris-HCl  (pH 8.0), 5 mM EDTA, 0.1% BSA (w/v), 0.1% β-ME (v/v)  C/D method:0.35 M sorbitol, 50 mM Tris-HCl (pH 8.0), 5 mM EDTA, 0.1% BSA, 0.1% β-ME (v/v) | | | | | | A/B/C/D method:  grinding, miracloth | | | | A method:  200 g/20 min  B method: 3000 g/10 min  C/D method: 1000 g/20 min | | | | A method: 3500 g/20 min, 3750 g/20 min  B method: 3000 g/10 min  C/D method: 30% / 52% sucrose gradient  (25000 rpm/1 h) | | | | | A method: 8 mL (10 mM NaCl, 100 mM Tris-HCl (PH 8.0), 50 mM EDTA, 1 mM DTT), 1.5 mL 20% SDS, 20 mL β-ME, 30 mL Proteinase K (10 mg/mL), 1.5 mL 5M KAc (PH 5.2)  B/C method:1% CTAB  D method: 20 uL DNAse I (10 mg/mL), 250 uL 200 mM MgCl_2_, 1% CTAB | | | | | | | A/B/C/D method: Phenol/Chloroform/Isoamyl-alcohol (25:24:1) (10000 g/20 min, Isopropyl alcohol (10000 g/20 min) | | | Shi et al. [44] |
| 1. *angustifolia, P. patula, A. bidwilli* | A: Isolation buffer A  B: Isolation buffer B  C: Isolation buffer C | A/B/C method: grinding, miracloth | A: 200 g/15 min, 3000 g/20 min  B: 200 g/20 min, 3500 g/20 min  C: 200 g/15 min, 2000 g/20 min | | | | | | | A: 30%/70% Percoll gradient (5000 g/25 min)  B/C 30%/52% sucrose gradient (3500 g/60 min) | | | | | | | | | | Lysis buffer: 8 mL (100 mM NaCl, 100 mM Tris-HC l(pH 8.0), 50 mM EDTA, 1 mM DTT), 1.5 mL 20% SDS,20 uL 2-ME, 30 uL proteinase K (10 mg/mL), 1.5 mL 5M KAc (pH 5.2) | | | | | | | | 10000 g/15 min, chloroform/isoamyl-alcohol (24:1) | | Vieira et al. [45] |
| *C. reinhardtii* | 0.3 M sorbitol, 50 mM HEPES-KOH (pH 7.5), 2 mM Na_2_-EDTA(pH 8.0), 1 mM MgCl_2_, 1% BSA | | | | | | | | | | | | | | | 0.35/0.55/0.75 MPa | | | | | | | 750 g/2 min | | 20%/45%/65% Percoll gradient (4200 g/15 min) | | | | | Quick et al. [46] |

| Continued to table S1 | | | | | | | | | | | | |
| --- | --- | --- | --- | --- | --- | --- | --- | --- | --- | --- | --- | --- |
| *rice* | LN method : 50 mM Tris-HCl ( pH 8.0), 0.35 M sucrose, 7 mM  EDTA, 5 mM 2- ME, 0.1% BSA  HS method: 1.25 M NaCl, 0.25 M ascorbic acid, 10 mM sodium metabisulfite, 12.5 mM borax, 50 mM Tris-HCl (pH 8.0), 7 mM EDTA, 1%(w/v) PVP-40, 0.1% (w/v)BSA, 1 mM DTT  PG method: 50 mM HEPES-KOH (pH 7.5), 0.33 M sorbitol, 5 mM MgCl_2_, 5 mM MnCl_2_, 5 mM EDTA,50 mM sodium ascorbate | | | LN/HS/PG method:  grinding, miracloth | | LN method : 1000 g/10 min  HS method: 200 g/20 min, 3500 g/20 min  PG method: 80% (v/v) Percoll gradient (2000 g/4 min) | | LN method :20%/45% discontinuous sucrose gradient (2000 g/30 min)  HS method: 1.25 M NaCl, 12.5 M borax, 1% (w/v), PVP-40, 50 mM Tris-HCl (pH 8.0), 25 mM EDTA, 0.1%(w/v) BSA, 1 mM DTT, 3500 g/20 min  PG method: 40%/80% discontinuous Percoll gradient (4000 g/10 min) | | LN method: CTAB, method/DNeasy Plant Mini Kit  HS method: DNeasy Plant Mini Kit  PG method: DNeasy Plant Mini Kit | | Takamatsu et al. [47] |
| *Festuca grass species* | Chloroplast Isolation Kit | grinding, cotton cloth | 200 g/7 min, 1300 g/7 min | | | | 30% Percoll gradient (1300 g/20 min) | | AP1 buffer with RNAse A | | DNeasy Plant Mini Kit | Islam et al. [48] |
| *Arabidopsis* | 50 mM HEPES/KOH, 0.33 M sorbitol, 1 mM MgCl_2_,  1 mM MnCl_2_, 2 mM EDTA (pH8.0), 5 mM Na-ascorbate, 0.1% BSA | | | | grinding | | 2000 g/2 min | | 40%/90% Percoll gradient (2000 g/10 min) | | | An et al. [49] |

'- ' means the result is available in article but there is no detail.
